# Neurophysiological Defects and Neuronal Gene Deregulation in *Drosophila mir-124* Mutants

**DOI:** 10.1371/journal.pgen.1002515

**Published:** 2012-02-09

**Authors:** Kailiang Sun, Jakub Orzechowski Westholm, Kazuya Tsurudome, Joshua W. Hagen, Yubing Lu, Minoree Kohwi, Doron Betel, Fen-Biao Gao, A. Pejmun Haghighi, Chris Q. Doe, Eric C. Lai

**Affiliations:** 1 Department of Developmental Biology, Sloan-Kettering Institute, New York, New York, United States of America; 2 Neuroscience Program, Weill Graduate School of Medical Sciences, Cornell University, New York, New York, United States of America; 3 Department of Physiology, McGill University, Montreal, Canada; 4 Department of Neurology, University of Massachusetts Medical School, Worcester, Massachusetts, United States of America; 5 Institute of Molecular Biology, University of Oregon, Eugene, Oregon, United States of America; 6 Department of Medicine, Weill Cornell Medical School, New York, New York, United States of America; 7 Institute of Computational Biomedicine, Weill Cornell Medical School, New York, New York, United States of America; Indiana University, United States of America

## Abstract

miR-124 is conserved in sequence and neuronal expression across the animal kingdom and is predicted to have hundreds of mRNA targets. Diverse defects in neural development and function were reported from miR-124 antisense studies in vertebrates, but a nematode knockout of *mir-124* surprisingly lacked detectable phenotypes. To provide genetic insight from *Drosophila*, we deleted its single *mir-124* locus and found that it is dispensable for gross aspects of neural specification and differentiation. On the other hand, we detected a variety of mutant phenotypes that were rescuable by a *mir-124* genomic transgene, including short lifespan, increased dendrite variation, impaired larval locomotion, and aberrant synaptic release at the NMJ. These phenotypes reflect extensive requirements of miR-124 even under optimal culture conditions. Comparison of the transcriptomes of cells from wild-type and *mir-124* mutant animals, purified on the basis of *mir-124* promoter activity, revealed broad upregulation of direct miR-124 targets. However, in contrast to the proposed mutual exclusion model for miR-124 function, its functional targets were relatively highly expressed in miR-124–expressing cells and were not enriched in genes annotated with epidermal expression. A notable aspect of the direct miR-124 network was coordinate targeting of five positive components in the retrograde BMP signaling pathway, whose activation in neurons increases synaptic release at the NMJ, similar to *mir-124* mutants. Derepression of the direct miR-124 target network also had many secondary effects, including over-activity of other post-transcriptional repressors and a net incomplete transition from a neuroblast to a neuronal gene expression signature. Altogether, these studies demonstrate complex consequences of miR-124 loss on neural gene expression and neurophysiology.

## Author Summary

microRNAs are abundant ∼22 nucleotide RNAs inferred to mediate pervasive post-transcriptional control of most genes. Still, relatively little is understood about their endogenous requirements and impact, especially in animal systems. We analyzed a knockout of *Drosophila mir-124*, which is conserved in sequence and neuronal expression across the animal kingdom, and predicted to have hundreds of mRNA targets. While dispensable for gross neural specification and differentiation, deletion of *mir-124* caused short lifespan, increased variation in dendrite numbers, impaired larval locomotion, and aberrant synaptic release at the NMJ. These phenotypes reflect extensive requirements of miR-124 even under optimal culture conditions. Loss of miR-124 broadly upregulated its direct targets but did not support the proposed mutual exclusion model, as its functional target genes were relatively highly expressed in neurons. One notable aspect of the direct miR-124 network was coordinate targeting of five positive components in the retrograde BMP signaling pathway, whose activation in neurons phenocopies loss of miR-124. Derepression of the direct miR-124 target network had many secondary effects, including over-activity of other post-transcriptional repressors and impaired transition from neuroblast to neuronal transcriptome signatures. Altogether, we demonstrate complex requirements for this conserved miRNA on gene expression and neurophysiology.

## Introduction

microRNAs (miRNAs) are ∼22 nucleotide (nt) regulatory RNAs that function primarily as post-transcriptional repressors. In animals, miRNAs have propensity to target mRNAs via 6–7 nt motifs complementary to their 5′ ends, termed “seed” regions [1]–[4]. This limited pairing requirement has allowed most miRNAs to capture large target networks. Analysis of multigenome alignments indicates that typical human miRNAs have hundreds of conserved targets, and that a majority of protein-coding genes are under miRNA control [5], [6]. The extraordinary breadth of animal miRNA:target networks has been extensively validated by transcriptome and proteome studies [7].

miR-124 is strictly conserved in both primary sequence and spatial expression pattern, being restricted to the nervous system of diverse metazoans, including flies [8], nematodes [9], *Aplysia* [10], and all vertebrates studied [11]–[13]. Such conservation implies substantial functions of miR-124 in controlling neural gene expression. miR-124 has been a popular model for genomewide investigations of miRNA targeting principles. For example, studies of miR-124 yielded the first demonstration of the downregulation of hundreds of direct targets detected by transcriptome analysis, and that this activity was driven by the miRNA seed region [14]. In addition, miR-124 provided one of the first illustrations of spatially anticorrelated expression of a miRNA and its targets [15], and was exploited for analysis of Ago-bound target transcripts [16]–[19] and direct identification of Ago-bound target sites [20].

Functional studies have connected vertebrate miR-124 to various aspects of neural specification or differentiation. Studies in chick ascribed miR-124 as a proneural factor that inhibits the anti-neural phosphatase *SCP1* [21]. However, no substantial effect of miR-124 on chick neurogenesis was found in a parallel study [22], although miR-124 was observed to repress neural progenitor genes such as *laminin gamma1* and *integrin beta1*. In the embryonic mammalian brain, miR-124 was reported to direct neural differentiation by targeting *polypyrimidine tract binding protein 1* (*PTBP1*), a global repressor of alternative splicing in non-neural cells [23]. In the adult mammalian brain, miR-124 promoted neural differentiation of the immediate progenitors, the transit-amplifying cells (TAs). Here, miR-124 directly targets the transcription factor *Sox9*, which maintains TAs and is downregulated during neural differentiation [24]. Other mammalian studies bolster the concept that miR-124 promotes neurogenesis [25] or neural differentiation [26]. One mechanism involves direct repression by miR-124 of *Baf53a*, a neural progenitor-specific chromatin regulator that must be exchanged for a neural-specific homolog to consolidate neural fate [27]. However, complicating the picture is the recent report that *Xenopus* miR-124 represses neurogenesis by directly targeting the proneural bHLH factor *NeuroD1* [28].

All vertebrate miR-124 loss-of-function studies have relied on antisense strategies and have yet to be validated by bona fide mutant alleles. However, as the three vertebrate *mir-124* loci are co-expressed in the nervous system, analysis of the null situation will require a triple knockout. So far, a *mir-124* knockout has only been described in *C. elegans*, which harbors a single copy of this gene [29]. Like most other miRNA mutants in this species, the loss of miR-124 did not cause obvious developmental, physiological or behavioral phenotypes. Nevertheless, comparison of gene expression in *mir-124*-expressing cells from wildtype and *mir-124* mutant animals revealed strong enrichment in miR-124 target sites amongst upregulated transcripts, revealing the impact of miR-124 on neuronal gene expression [9]. The broad, but phenotypically-tolerated, misregulation of miR-124 targets in this species is potentially consistent with the “fine-tuning” model for miRNA regulation.

Here, we analyze a knockout of the sole *mir-124* gene in *D. melanogaster*. Although this mutant is viable and exhibits grossly normal patterning, we documented numerous phenotypes, including short lifespan, increased variation in the number of dendritic branches of sensory neurons, decreased locomotion and aberrant synaptic release at CNS motoneuron synapses. All of these phenotypes were rescued by a single copy of a 19 kilobase (kb) genomic transgene encompassing the *mir-124* locus. We generated a transcriptional reporter of *mir-124* that recapitulated the CNS expression of endogenous *pri-mir-124*, and used this to purify *mir-124*-expressing cells from stage-matched wild-type and *mir-124*-mutant embryos. Transcriptome analysis revealed strong enrichment of direct miR-124 targets amongst genes upregulated in *mir-124*-mutant cells. The miR-124 target network included coordinate repression of multiple components in the retrograde BMP signaling pathway, whose activity controls synaptic release. Loss of miR-124 further correlated with increased activity of other neural miRNAs and the neural translational regulator Pumilio, and had the net effect of impairing transition from the neuroblast to neuronal gene expression signature. Altogether, we demonstrate that endogenous miR-124 has substantial impact on CNS gene expression, which underlie its requirement for organismal behavior and physiology.

## Results

### Neural expression of *Drosophila mir-124*

**Figure 1:**
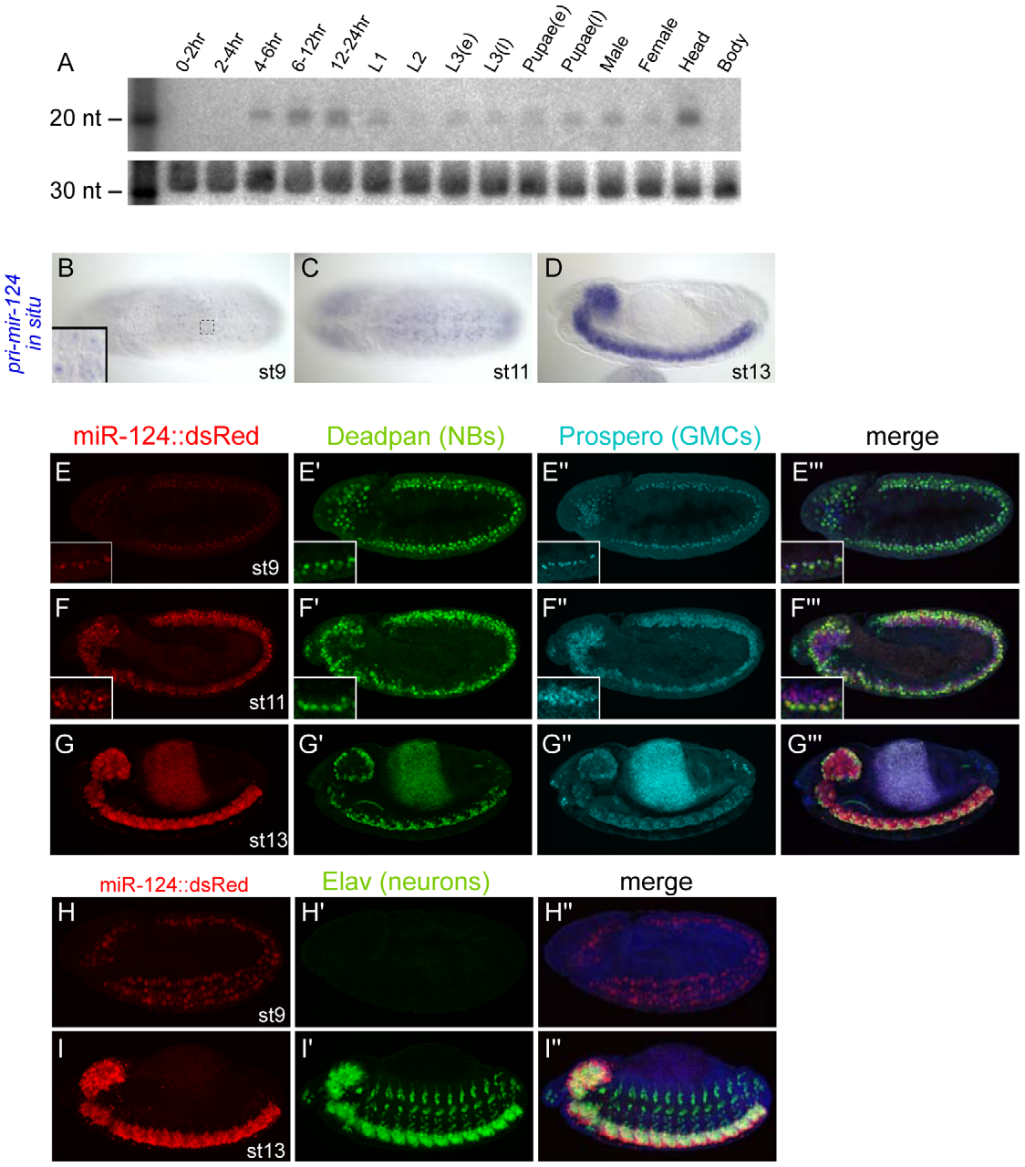
Temporal and spatial expression of *Drosophila* miR-124. (A) Northern analysis using staged preparations of total RNA. (B–D) Nascent transcription of *pri-mir-124* detected with a 1 kb probe. (B and C) are ventral views and (D) is a lateral view. Inset of panel (B) highlights the detection of nuclear dots that reflect the chromosomal locations of *mir-124* transcription. (E–I) Expression of a miR-124:DsRed transgene colabeled with various neural markers, Deadpan (neuroblast marker) and Prospero (ganglion mother cell marker) and Elav (differentiated neuron marker); embryos in H–I are counterstained with DAPI. In all panels, miR-124:DsRed is at left, the neural markers in the middle, and merged images at right; the signal in the center of panels G is gut autofluorescence. Activity of *mir-124* initiates in neuroblasts and is maintained in GMCs and CNS neurons. High magnification insets of panels E–F show gradual expression of miR-124:DsRed in all Deadpan+ and Prospero+ positive cells in CNS.

Northern analysis first detected mature miR-124 at 4–6 hrs of development (Figure 1A), corresponding approximately to embryo stages 9–10. Its level peaked during 12–24 hrs, declined during the first and second larval stages, and was then upregulated in the third instar through adulthood. The apparent temporal fluctuation in miR-124 levels appeared to be a consequence of its tissue-specificity. For example, most miR-124 in the adult was present in the head (Figure 1A), consistent with comparison of head and body small RNA data [30]. We therefore used *in situ* hybridization to primary miRNA transcripts to analyze expression of *Drosophila mir-124* at the cellular level [8]. Close examination showed that its primary transcription, as reflected by nuclear dots of elongating *pri-mir-124* transcripts (Figure 1B, inset), was first detected in the ventral nerve cord around stage 8 during germband elongation (Figure S1) and became more prominent in subsequent stages. Its expression in the ventral nerve cord and brain was maximal in the fully germband retracted embryo from stage 13 onwards (Figure 1B–1D).

To facilitate analysis of *mir-124* expression, we generated a transcriptional reporter. We fused 4.2 kb of sequence upstream of the *mir-124* hairpin, including ∼1 kb more genomic sequence than the previously studied *mir-124:Gal4* transgene [31], to a nuclear DsRed gene in the insulated H-Red-Stinger vector. Multiple transgenic lines exhibited identical expression in the embryonic nervous system that recapitulated endogenous *pri-mir-124* expression. Similar to endogenous *pri-mir-124*, the *mir-124:DsRed* transgene was faintly active at stage 8 (Figure S1), and exhibited nearly completely colocalization with the pan-neuroblast marker Deadpan in the stage 9 CNS (Figure 1E, 1E′); at this stage mature neurons have not yet been specified. Neuroblasts (NBs) divide to regenerate the NB as well as a ganglion mother cell (GMC). GMCs can be marked by Prospero, and these cells were similarly labeled by *mir-124:DsRed* (Figure 1E, 1E″). We continued to observe DsRed expression in NBs and GMCs as development proceeded (Figure 1F, 1G). GMCs divide to generate sibling cells and neurons, and neuronal commitment is marked by expression of Elav. *mir-124:DsRed* was active in the full complement of neurons in the CNS, but Elav alone was highly expressed in the peripheral nervous system (Figure 1H, 1I).

### Generation of *mir-124* knockout and genomic rescue strains

**Figure 2:**
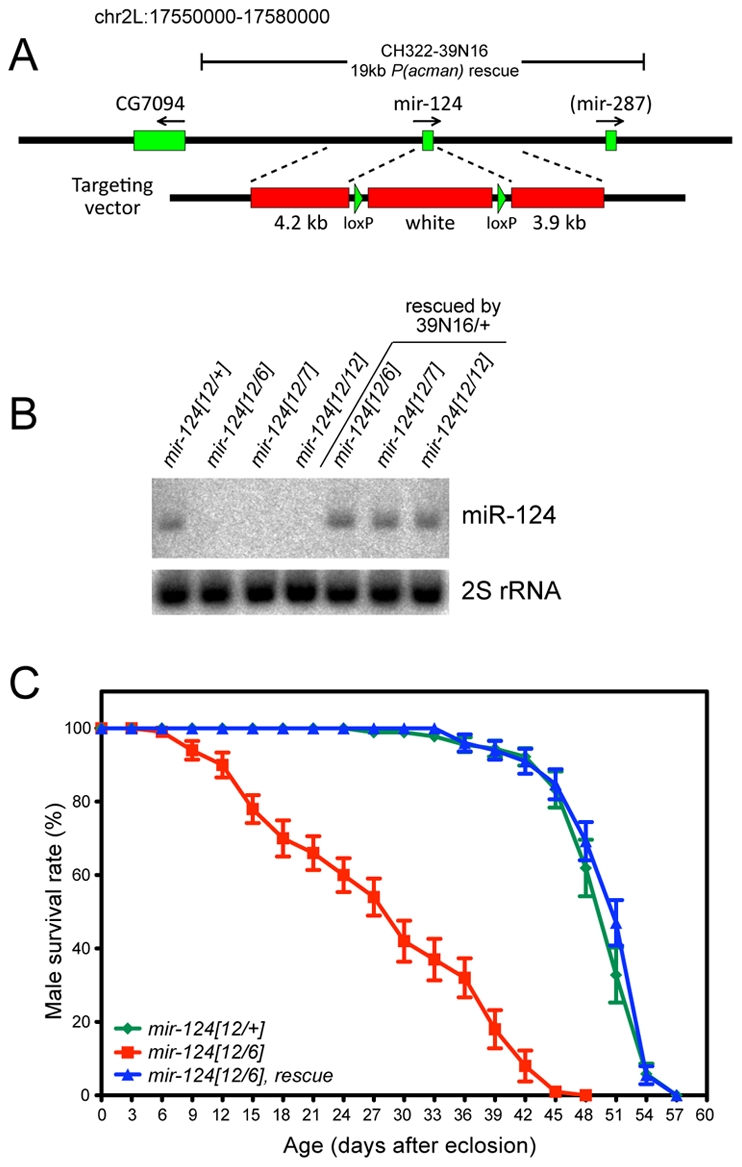
General characterization of *mir-124* knockout and rescue strains. (A) The *pre-mir-124* hairpin was replaced with *mini-white+* using ends-out homologous recombination. A 19 kb *mir-124* rescue transgene lacks known protein-coding genes; it overlaps *mir-287* but this locus has not been validated as a miRNA from deep sequencing [32]. (B) Northern validation that adult *mir-124* knockouts are null for mature miR-124; a normal level of miR-124 is restored by the rescue transgene. (C) The substantially shortened lifespan of *mir-124* transheterozygous males raised at 29°C was fully rescued by the *mir-124* transgene.

We used ends-out homologous recombination to replace the endogenous *mir-124* hairpin with a *white+* marker flanked by loxP sites (Figure 2A). We established several knockouts from independent insertions of the original targeting vector, so that we could query trans-heterozygous deletion combinations. We also deleted the *white+* marker to obtain clean deletions of the locus. As these behaved similarly to the *white+* alleles (not shown), most subsequent analyses utilized the latter alleles since the marker facilitated the construction of recombinant lines. We used Northern analysis to verify that multiple independent *mir-124* knockout alleles did not express mature miR-124 (Figure 2B), demonstrating that these are truly null backgrounds.

The *mir-124* mutant alleles were viable and fertile, and exhibited normal external morphology. However, they were not easily kept as homozygous stocks, potentially reflecting detrimental effects of *mir-124* deletion. Because homologous recombination in *Drosophila* can induce unlinked aberrations, which might theoretically be shared by independent targeting events, we were cautious in the comparison of trans-heterozygous mutants to wildtype. We therefore generated a P[acman] insertion of 19 kb of *mir-124* genomic DNA (*39N16*, Figure 2A), a region lacking annotated protein-coding genes; note that it contains *mir-287*, but this locus has not been confirmed in largescale sequencing [30], [32]. We recombined the 39N16 rescue with *mir-124* deletion alleles, and used Northern analysis to validate that this transgene restored a normal level of miR-124 to mutant adults (Figure 2B). We subsequently focused on phenotypes evident in trans-heterozygous animals compared to heterozygotes, that were rescued by the *mir-124* genomic transgene.

We observed that 60–70% of *mir-124* deletion embryos of various genotypes failed to hatch, and that embryonic lethality was substantially (although not fully) rescued by the *mir-124* genomic transgene (Figure S2). Following embryogenesis, we did not observe substantial differences in viability between the *mir-124* mutant and wildtype, at larval/pupal/adult stages (Figure S2). However, *mir-124* mutant adult males exhibited substantially shortened lifespan, and this defect was completely rescued by introduction of the *mir-124* genomic transgene (Figure 2C). These observations suggest that miR-124 is detectably required for organismal fitness.

### Lack of strong defects in neural production or differentiation in *mir-124* mutants

**Figure 3:**
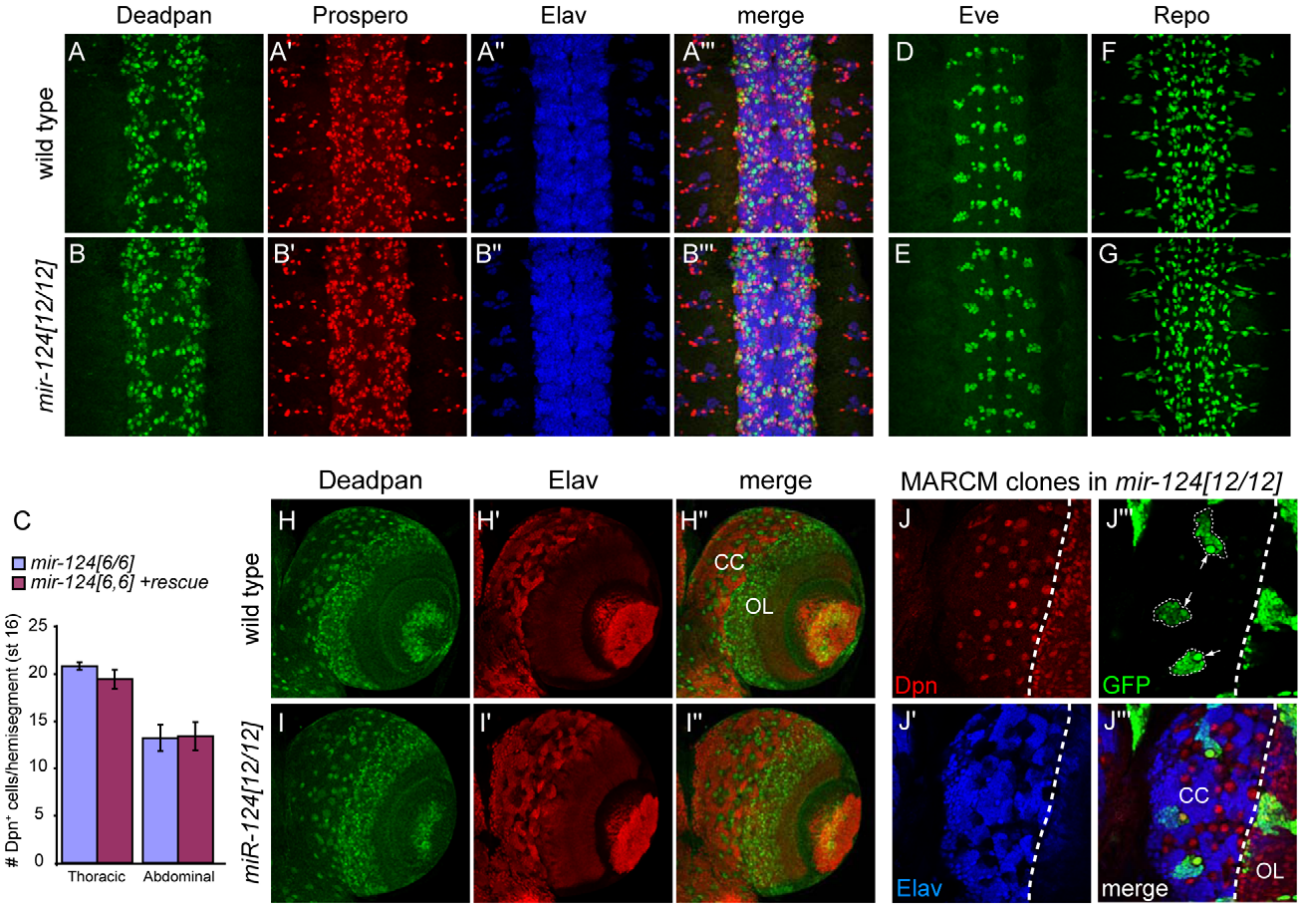
Absence of major defects in specification of the nervous system of *mir-124* mutants. (A–F) Stage ∼13 embryos, ventral aspect. A and B are triple labelings of Deadpan (NB), Prospero (GMC) and Elav (neuron); no substantial differences were observed. (C) Graph indicates the number of Dpn+ cells per hemisegment comparing *mir124[6/6]* embryos to mutants carrying the genomic rescue. Error bar represents standard deviation from the average of five embryos; 20 and 30 hemisegments were quantified for the thoracic and abdominal segments, respectively. Since subtler differences might not be seen with pan-neuronal labeling, we analyzed Eve (D, E), which is active in a subset of CNS neurons and sibling cells; the mutant was similar to wildtype. (F, G) Expression of the glial marker Repo was not markedly different in *mir-124* mutants. (H–J) Larval brains. (H–I) Specification of neuroblasts and neurons is relatively similar in wildtype and *mir-124* mutant. (J) MARCM analysis in *mir-124* mutant brain to mark the lineages produced by single neuroblasts. GFP+ mutant clones maintain a single neuroblast (marked by large Dpn+ cells, arrows in J″) and can generate multiple neurons.

Because of the specific expression of *mir-124* in the CNS, we were interested to see if we could uncover any defects in neural development. We analyzed a number of CNS markers, but did not detect obvious changes across a panel of neuroblast and GMC markers, including Deadpan and Prospero (Figure 3A, 3B) and Hunchback and Miranda (Figure S3). Careful quantification of the numbers of Deadpan+ neuroblasts did not reveal differences within either thoracic or abdominal segments (Figure 3C). The overall pattern of Elav was also normal (Figure 3A, 3B). Since many cells express Elav, we also checked Even-skipped, which is expressed in small populations of neurons and sibling cells, but these also appeared relatively normal (Figure 3D, 3E and Figure S3). We further analyzed the glial marker Repo, which was reported as a direct miR-124 target with anti-correlated expression [15], [33], but its pattern was not substantially altered (Figure 3F, 3G). Finally, *mir-124* mutants exhibited grossly normal axonal architecture in the late embryo, as marked by 22C10 (Figure S4).

To assess a possible phenotype in later development, we also examined the larval CNS. We detected abundant activity of *mir-124:DsRed* in the larval CNS, including both the brain and ventral nerve cord (Figure S5A). Within the brain, activity or *mir-124:DsRed* was highest in the central complex (Figure S5B). However, Deadpan/Elav staining showed relatively normal patterns of neuroblasts and neurons in the *mir-124* mutant brain (Figure 3H, 3I). Finally, we assessed the proliferation of larval neuroblast clones using the MARCM technique. Using this strategy, the neural progeny of single neuroblasts can be labeled *in situ* (Figure S5C). We observed that *mir-124* mutant neuroblast clones appropriately maintained a single neuroblast and could undergo multiple divisions to generate many neurons (Figure 3J). This level of analysis does not address potential quantitative defects in neuroblast clones, nor does it rule out that a subpopulation of cells may have developed abnormally. However, pan-CNS *Drosophila* miR-124 does not appear to be required for bulk aspects of neurogenesis or differentiation, as has been concluded for its vertebrate counterparts.

### Specific behavioral and electrophysiological defects in *mir-124* mutants

**Figure 4:**
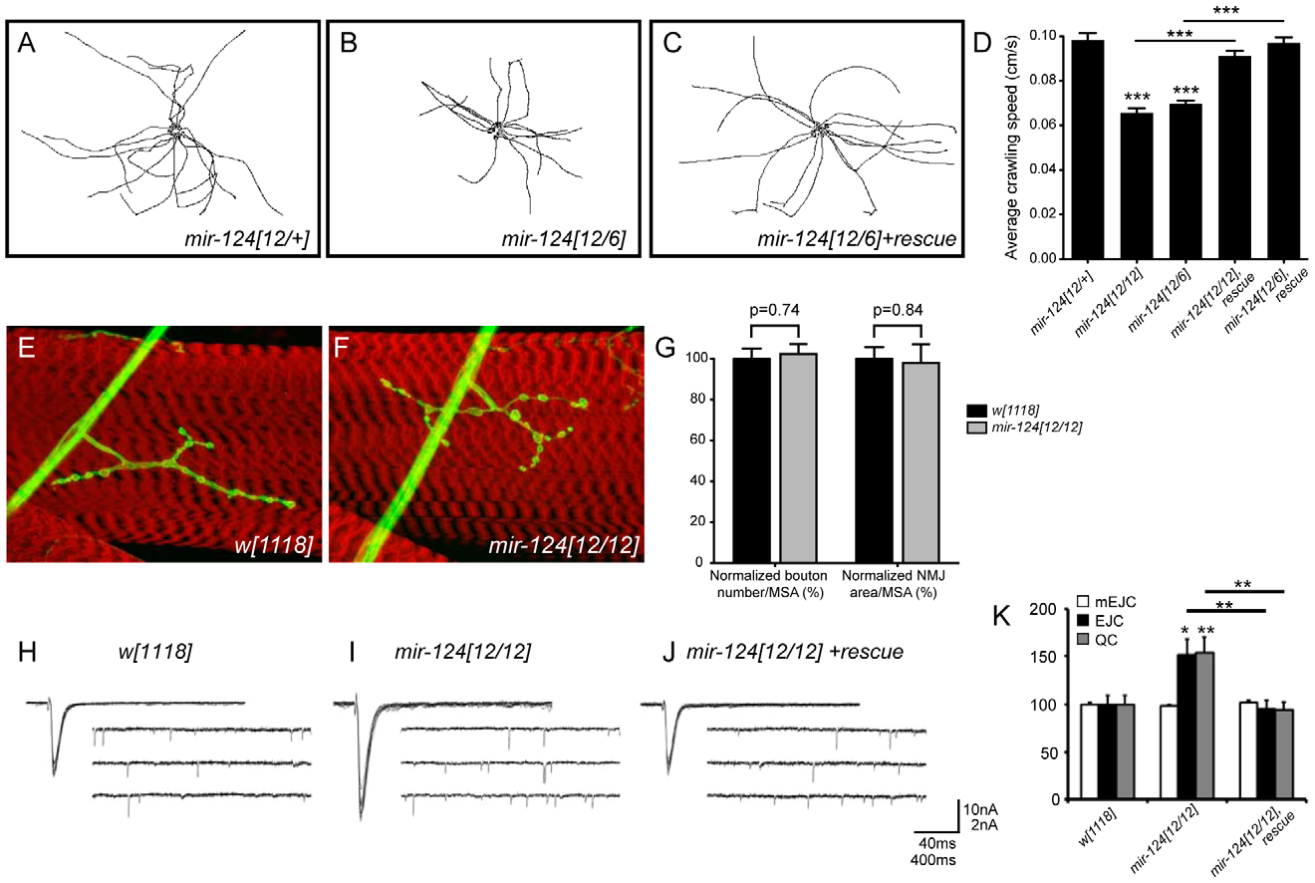
Requirement of *mir-124* for larval locomotion and synaptic transmission. (A–C) Locomotion defects in *mir-124* mutants. Each track depicts the movement of an individual 3rd instar larva tracked for one minute; 15 tracks were superimposed to reveal population behavior. (D) Quantitative analysis showed that both *mir-124* mutant genotypes exhibited substantially reduced locomotion, which was rescued by *mir-124* genomic transgene. (E, F) HRP staining (green) of the neuromuscular junction on muscle 4 (counterstained in phalloidin) in wildtype and *mir-124* mutant. (G) Quantitative analysis (n = 12) showed no significant difference in bouton numbers or NMJ area, as normalized to muscle surface area (MSA). (H–K) Loss of miR-124 leads to an enhancement of presynaptic neurotransmitter release. (H–J) Representative traces of evoked (excitatory junction currents, EJC) and spontaneous (miniature EJC, mEJC) membrane currents recorded from muscle 6 in the third abdominal segment in wandering third-instar larvae of *w[1118]*; *mir-124[12/12]* and *mir-124[12/12]* rescued by genomic insert. EJC contain 10 consecutive superimposed traces and mEJC are three traces of continuous recordings. (K) Quantification of mEJC, EJC, and quantal content (QC) for the indicated genotypes. Deletion of *mir-124* resulted in no differences in spontaneous activity, but caused significant increases in evoked currents and quantal content; these phenotypes were rescuable. n = 22 NMJs for each genotype. Error bars represent SEM, statistical tests by two-tailed t-test show *p<0.05, **p<0.01, ***p<0.001.

Since we did not observe substantial defects in neural development, we checked for functional defects in the central nervous system. An informative assay involved tracking the locomotion of third instar larvae. We examined the movements of cohorts of larvae in 1 minute movies, and quantified total distance traveled and crawling speed. Different trans-heterozygous *mir-124* mutant combinations exhibited a clear defect in both parameters, and these were fully rescued by the *mir-124* genomic transgene (Figure 4A–4C and Figure S6); the differences were highly statistically significant (Figure 4D). Therefore, miR-124 is required for normal locomotion.

To gain functional insight into the basis of this defect, we first tested for a possible role of miR-124 in synaptic structure. We analyzed the arborization of neuromuscular junctions (NMJs) of CNS motoneurons in third instar larvae (Figure 4E, 4F), but this did not reveal significant changes in the number of NMJ boutons or the area arborized (Figure 4G). We therefore went on to analyze the activity of these synapses. Using the two-electrode voltage clamp technique, we measured both spontaneous miniature excitatory junctional currents (mEJCs) and evoked junctional currents (EJCs) from control and *mir-124* mutant larvae (Figure 4H–4J). mEJCs were indistinguishable in the two groups, but *mir-124* mutants showed an increase in the average EJC amplitudes, indicating a significant elevation in quantal content at the NMJ (Figure 4K). The increase in EJCs was fully rescued when we included a *mir-124* genomic transgene in the homozygous mutant larvae, indicating that the increase in EJCs and quantal content was attributable to the *mir-124* deletion. Therefore, miR-124 serves to limit synaptic activity.

### miR-124 reduces the variability of dendritic numbers of sensory neurons

**Figure 5:**
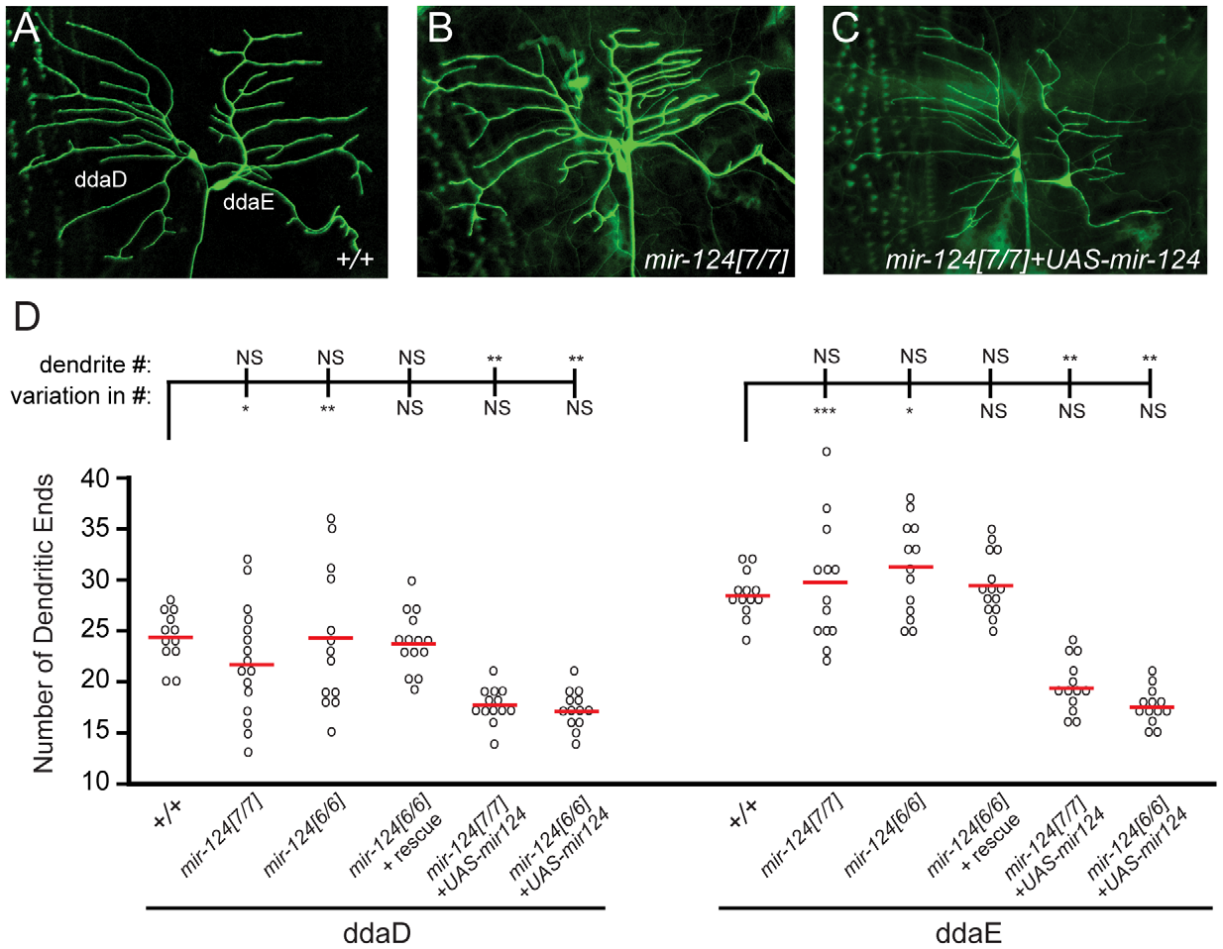
*mir-124* suppresses variation in dendrite numbers on sensory neurons. (A–C) We labeled ddaD and ddaE neurons with CD8-GFP under the control of *Gal4^221^*. Representative images are shown from *mir-124* loss- and gain-of-function backgrounds. The overall patterns of dendrite branching in wildtype (A) and mutant (B) were similar. (C) Misexpression of *mir-124* in the *mir-124* mutant strongly decreased the complexity of dendritic branching. (D) Quantitative analysis. Each of the circles represents dendrite quantification of an individual neuron. Although the average numbers of dendritic ends for ddaD and ddaE neurons in *mir-124[6]* or *mir-124[7]* mutants were not statistically different those in wildtype, the variation in their numbers was significantly increased (by F test). The effect was more pronounced in ddaD than ddaE neurons, but both were rescued by the *mir-124* genomic transgene. Analysis of *mir-124* overexpression in the *mir-124* mutant background showed a strong decrease in branching complexity; still, the variation in dendritic end numbers was rescued.

Expression of *Drosophila mir-124* was confidently detected only in CNS, but did not exclude potential expression in the PNS. Of note, *C. elegans mir-124* is predominantly expressed in sensory neurons [9]. We therefore checked for PNS phenotypes, judging that defects that were rescuable should reflect endogenous requirements for miR-124. This analysis revealed a defect in the differentiation of dendrites in a subset of sensory neurons (Figure 5A–5C). On the one hand, the average number of dendritic branches in *mir-124* mutants did not show a statistical difference from that in wildtype larvae. However, the variation in dendrite numbers was substantially increased in *mir-124* mutants (Figure 5D); this was especially noticeable for ddaD. This defect was rescued by the *mir-124* genomic transgene, indicating that miR-124 suppresses variability in dendritic branching numbers.

We also tested the effect of misexpressing miR-124 in class I neurons, building on our observation that ectopic miR-124 reduces dendrite numbers in wild-type [31]. Misexpression of miR-124 in *mir-124* mutants, using a newly constructed *UAS-DsRed-mir-124* transgene, recapitulated this defect (Figure 5C). Despite the gain-of-function phenotype of reduced dendrite number, the variation in dendrite numbers across the population was rescued (Figure 5D). These results suggest that miR-124 helps maintain the consistency of dendritic branching patterns of specific neurons. Such a function has not yet been reported from the study of other dendrite mutants [34].

### Cell-autonomous misregulation of direct targets in *mir-124* mutants

**Figure 6:**
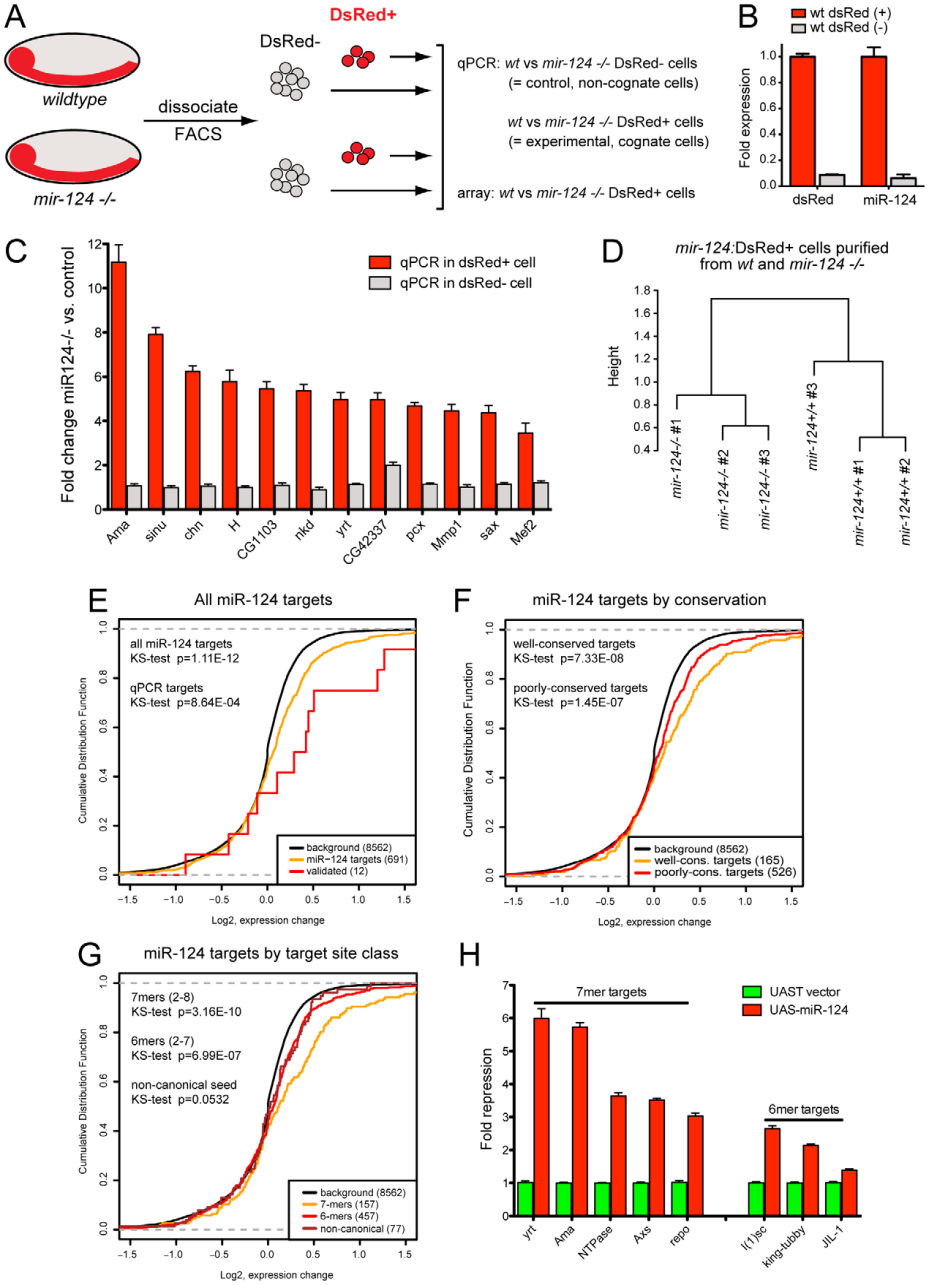
Gene expression in miR-124+ cells from wild-type and *mir-124* mutants. (A) Scheme for isolation and analysis of cells. (B) qPCR validation that the sorted DsRed+ cells specifically express *mir-124*. (C) qPCR analysis of predicted miR-124 targets showed upregulation in miR-124:DsRed+ cells, but not miR-124:DsRed- cells. (D) Unsupervised hierarchical clustering of microarray data from miR-124:DsRed+ cells purified from wildtype and *mir-124* mutant embryos collected 10–16 hrs after egg laying. (E–G) Cumulative distribution function (CDF) plots of various sets of predicted miR-124 targets in mutant vs. wildtype microarray data. Shifts to the right reflect overall upregulation of genes in the *mir-124* mutant. (E) Global upregulation of all predicted miR-124 targets. (F) Transcripts with well-conserved miR-124 sites were upregulated more strongly than those with poorly-conserved target sites, although both sets were significantly upregulated. (G) Transcripts with 2–8 seed matches were upregulated more strongly than transcripts with 2–7 or non-canonical seed matches. (H) Sensor validation of direct repression of miR-124 targets by ectopic miR-124. Transcripts with 2–8 (7mer) targets generally repressed more strongly than those with 2–7 (6mer sites).

Having established a variety of clear phenotypes in *mir-124* mutants, we wished to query changes in gene expression in the mutant cells. Because this miRNA is only expressed in the nervous system, we did not expect to be able to make specific measurements using whole embryos. Instead, we took advantage of the *mir-124:DsRed* reporter to isolate *mir-124*-expressing cells from dissociated embryos using fluorescence activated cell sorting (FACS). We introduced *mir-124:DsRed* into the *mir-124* mutant background, so that we could isolate the relevant mutant cells (Figure 6A). Consistent with the lack of substantial neural specification defects in the mutant, the expression of the *mir-124* reporter was similar in the presence or absence of the miRNA. This suggested that transcriptional profiling by this strategy was not likely to be substantially affected by the absence of cell types whose specification might require miR-124, or that might fail to be isolated because of positive autoregulatory feedback of miR-124 onto its own transcription. We note that analogous *mir-124* promoter fusions in nematode and zebrafish were correctly expressed in the absence of endogenous *mir-124* and *Dicer*, respectively [9], [35].

Recognizing that substantial manipulation is incurred during embryo dissociation and cell sorting, we were interested to obtain confidence that potential changes in gene expression in our measurements could be specifically attributed to miR-124 activity. Although some degree of non-autonomous regulatory effect is plausible, for example due to miRNA targeting of signaling factors, a general expectation is that the direct regulatory effects of a miRNA should be cell autonomous. Therefore, we sought to gauge the specificity of gene expression changes by comparing cells that normally express the miRNA with those that do not.

To do so, we separated miR-124:DsRed+ and DsRed− cells from stage 13–16 embryos (∼10–16 hrs of development) that were wildtype or deleted for *mir-124*. We chose this as a temporal window that was late enough to permit the full pattern of miR-124 expression to be established, but putatively early enough to minimize highly indirect changes in gene expression (i.e., that might arise during the remainder of embryogenesis from 16–22 hrs). Post-sort analysis showed that ∼80% of the selected cells were DsRed+, and qPCR analysis of these sorted wild-type cells confirmed that the DsRed+ cells specifically expressed *pri-mir-124* (Figure 6B). We then examined a panel of transcripts with high-ranking TargetScan scores (http://www.targetscan.org/) and conserved miR-124 target sites in their 3′ UTRs (Figure 6C). We could indeed validate many such targets as being upregulated in miR-124:dsRed+ cells isolated from *mir-124* mutants by qPCR (Figure 6C). In contrast, we observed very few changes in these same transcripts in *mir-124* mutant cells that did not express miR-124:DsRed, indicating that their deregulation was likely a direct consequence of miR-124 activity.

### Transcriptome-wide derepression of miR-124 targets

With these data in hand, we moved to transcriptome-wide analysis. We purified three biologically independent samples of miR-124:DsRed+ cells from dissociated wildtype and *mir-124* mutant stage 13–16 embryos and profiled them using Affymetrix microarrays. We generated sufficient RNA from purified cells so that only a single amplification step was required. The triplicate wild-type and *mir-124*-mutant transcriptomes were highly segregated by unsupervised hierarchical clustering (Figure 6D), indicating that major changes in expression profiles were due to genotype and not to technical variation.

Although several genomewide studies in vertebrates demonstrated upregulation of direct targets upon miRNA depletion or knockout [36]–[39], in some cases a genomewide signature was not recovered with mutants of single-copy, tissue-specific, miRNAs (e.g. *mir-182*) [40]. Therefore, broad upregulation of targets in a miRNA mutant is not a given. We plotted the cumulative distribution function (CDF) of various sets of genes, comparing their levels in the *mir-124* mutant relative to wildtype. Indeed, transcripts bearing miR-124 sites predicted by mirSVR [41] exhibited a highly statistically significant shift to higher levels in *mir-124* mutants (*p*-value<1.11e-12) (Figure 6E); i.e. shifted to the right in the CDF plot. Therefore, endogenous miR-124 strongly influences the transcriptome of the *Drosophila* nervous system. Moreover, derepression of direct targets accounted for a substantial proportion of the most deregulated genes in *mir-124* mutants, since 24/59 genes upregulated >2-fold with p-value<0.05 bore miR-124 seed sites (Table S1).

We further divided targets into a poorly-conserved cohort (site alignment is confined, at most, to the five melanogaster group species) and a well-conserved cohort (target site is aligned in both melanogaster group and non-melanogaster group Drosophilids). As has been observed in vertebrate systems, well-conserved targets of fly miR-124 were overall repressed more potently than poorly-conserved targets (Figure 6F). Nevertheless, recently-evolved miR-124 target sites exerted palpable regulatory impact in the intact animal, since transcripts with such sites were detectably shifted in their expression relative to background. We also subdivided targets by category (7mer, 6mer and non-canonical sites with seed-mismatches) and observed that these conferred progressively less regulation (Figure 6G). To validate the capacity for direct targeting of these transcripts by miR-124, we assayed the response of luciferase-3′ UTR sensors to ectopic miR-124 in S2 cells. Analysis of 8 such sensors, bearing single conserved 7mer or 6mer sites, showed that all were significantly repressed upon transfection of *ub-Gal4 and UAS-mir-124* expression constructs (Figure 6H). The distribution of repression values confirmed that 7mers generally yielded greater repression than 6mers. In summary, this first transcriptome-wide analysis of target expression in purified cell populations in a *Drosophila* miRNA mutant supports general notions of target site activity from vertebrate studies.

### The expression of miR-124 is not mutually exclusive with its functional targets

A general principle of miRNA targeting emerged from comparing the spatial expression of tissue-specific *Drosophila* miRNAs with their predicted targets. A bias for spatial anti-correlation of such miRNAs and their targets was observed, termed “mutual exclusion” [15]. For example, neural genes were depleted of miR-124 target sites while epidermal genes were enriched for miR-124 target sites. Since all of these cell types derive from a common progenitor, the neuroectoderm, this led to the model that expression of miR-124 helps to repress epidermal potential in neurons [15].

**Figure 7:**
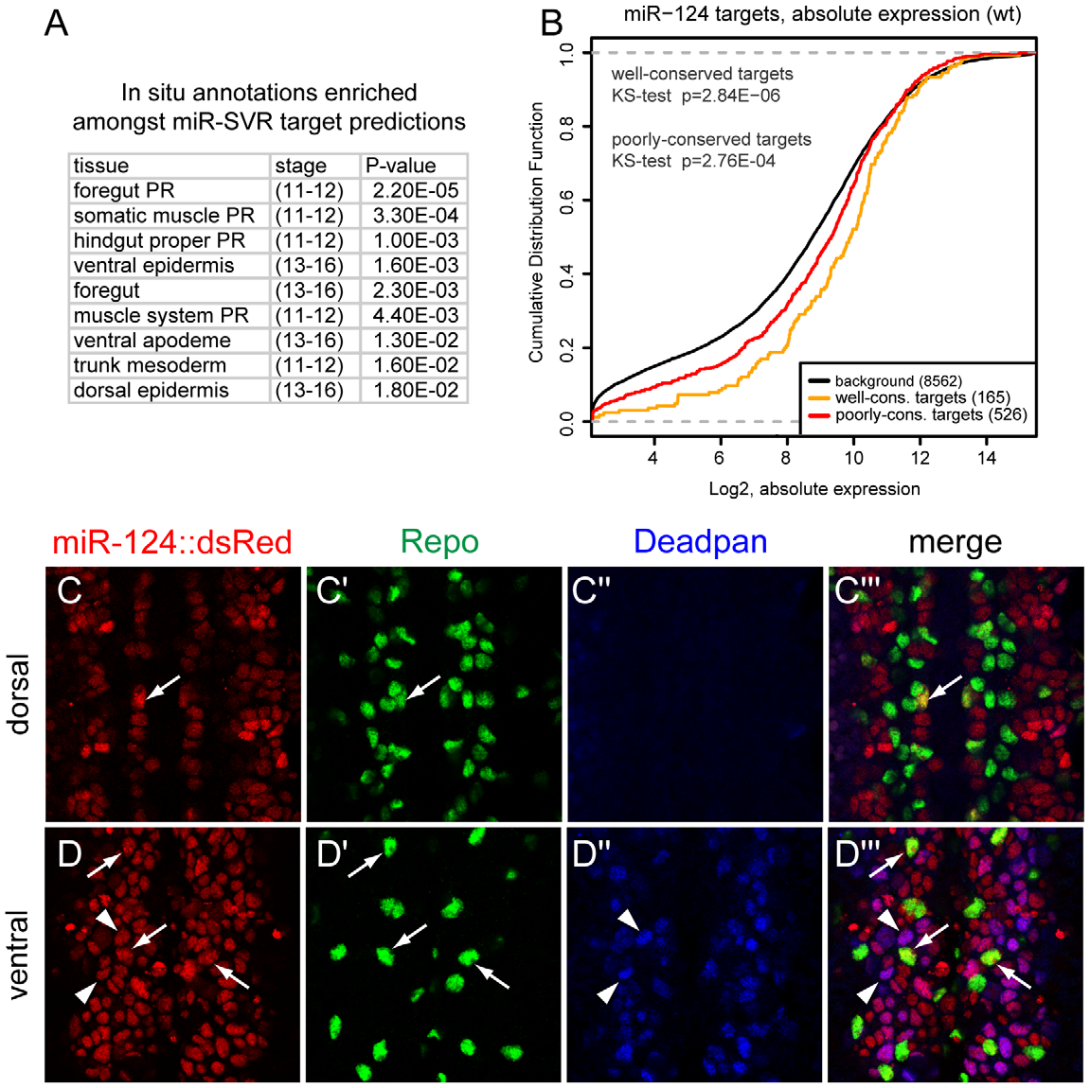
Lack of evidence for mutual exclusion amongst the functional miR-124 target network. (A) Consistent with earlier reports [15], transcripts bearing miR-124 target sites predicted by mirSVR are enriched for genes annotated with non-neural expression. The top enriched tissue annotations are shown in rank order. (B) The absolute levels of transcripts bearing miR-124 target sites in miR-124:DsRed+ cells are well above average gene expression. Moreover, transcripts bearing well-conserved sites are overall more highly expressed than those with poorly-conserved sites. (C, D) Triple label of stage 14 embryos for miR-124:DsRed, Repo (a glial marker) and Deadpan (a neuroblast marker). (C) In more dorsal planes lacking neuroblasts, miR-124:DsRed is excluded from most Repo+ cells, although rare cells show colocalization (arrow). (D) In more ventral planes containing NBs, miR-124:DsRed colocalizes with Deadpan+ cells (arrowheads) as well as Repo+ (arrows) cells inferred to be glioblasts.

In principle, such a pattern might reflect an active role of miR-124 to suppress the epidermal program in neurons, or might reflect a fail-safe program that is secondary to transcriptional mechanisms. We were in a position to test this using our gene profiling data from wildtype and mutant miR-124-expressing cells. We first tested whether we could reproduce the mutual exclusivity principle amongst miR-124 target genes, as defined by an independent set of miRNA target predictions generated using mirSVR [41] and multiZ alignments of twelve *Drosophila* genomes [42]. Together, this analysis incorporates more information on miRNA targeting and more genomes than were available earlier [15]. Indeed, cross-referencing these target predictions against *in situ* annotations catalogued from *Drosophila* embryogenesis [43] confirmed that epidermal genes were enriched amongst miR-124 targets at stages 11–12 and 13–16 (Figure 7A), as reported earlier [15].

However, when performing a similar analysis using our data from functional derepression in *mir-124* mutant cells, we failed to observe broad derepression of epidermal target genes, either amongst well-conserved or poorly-conserved target sets (Table S2). There were certainly individual miR-124 targets that are expressed and/or function in epidermal development, but this was not an overall trend amongst derepressed miR-124 targets (Figure 6E–6G). We also did not observe overall enrichment for epidermal genes amongst all upregulated genes (thus including both direct and indirect effects, Table S2), and only a few transcripts with miR-124 targets were absent in wild-type miR-124:DsRed+ cells and now present in *mir-124* mutant cells (17/204 putative targets, but only 2 of these bore conserved sites; Table S1). Overall, these observations suggested that mutual exclusion of miR-124 and target accumulation is not a feature actively driven by miRNA activity.

We investigated this further by examining the absolute levels of predicted miR-124 targets in miR-124-expressing cells. miR-124 targets exhibited a strong trend to be amongst the more highly expressed genes compared to non-targeted transcripts; this was true not only in the *mir-124* mutant but also in wildtype (Figure 7B and Figure S7). Moreover, well-conserved miR-124 targets were generally more highly expressed than poorly-conserved targets, even in wild-type miR-124-expressing cells (Figure 7B). We conclude that evolutionary selection of miR-124 target sites in miR-124-expressing cells is biased for transcripts that accumulate to above-average levels, even though the presence of miR-124 target sites clearly decreases the endogenous levels of these target transcripts (Figure 6E–6G).

To complement these quantitative data with cellular data, we examined the expression of the miR-124 target Repo [33], which we confirmed to be directly responsive to miR-124 (Figure 6H). The spatial expression of miR-124 and Repo was previously reported to be mutually exclusive [15], and we confirmed exquisite exclusion of their domains in the ventral ectoderm, where miR-124 is active in neurons and Repo in glia (Figure 7C–7C′″). Only in rare cells could we observe co-expression of these markers, and these might potentially be due to reporter perdurance. Looking more ventrally into the progenitor layer, we observed strong co-expression of miR-124:DsRed with neuroblasts marked by Deadpan (Figure 7D), as noted earlier (Figure 1E–1G). However, this layer also contained strongly Repo-positive cells (Figure 7D′) that colabeled with miR-124:DsRed but were exclusive of Deadpan; we infer these to be glioblasts. As these cells are progenitors, perdurance does not appear to explain co-expression of miRNA reporter and target. We infer that a phase of coexpression of miR-124 and *repo* precedes the adoption of their mutually-exclusive state.

Overall, these data indicate a substantial trend for co-expression of miR-124 and its targets genomewide, as similarly deduced from studies of miR-124 in zebrafish [35] and *C. elegans* [9]. Furthermore, while we could confirm that mutual exclusion with epidermal genes is clearly a feature of the target network selected by *Drosophila* miR-124, it does not seem to be a major determinant in directing neuronal-specific programs of gene expression, since epidermal genes were not overall substantially upregulated in the absence of the miR-124.

### Coordinate targeting of retrograde BMP signaling components by miR-124

**Figure 8:**
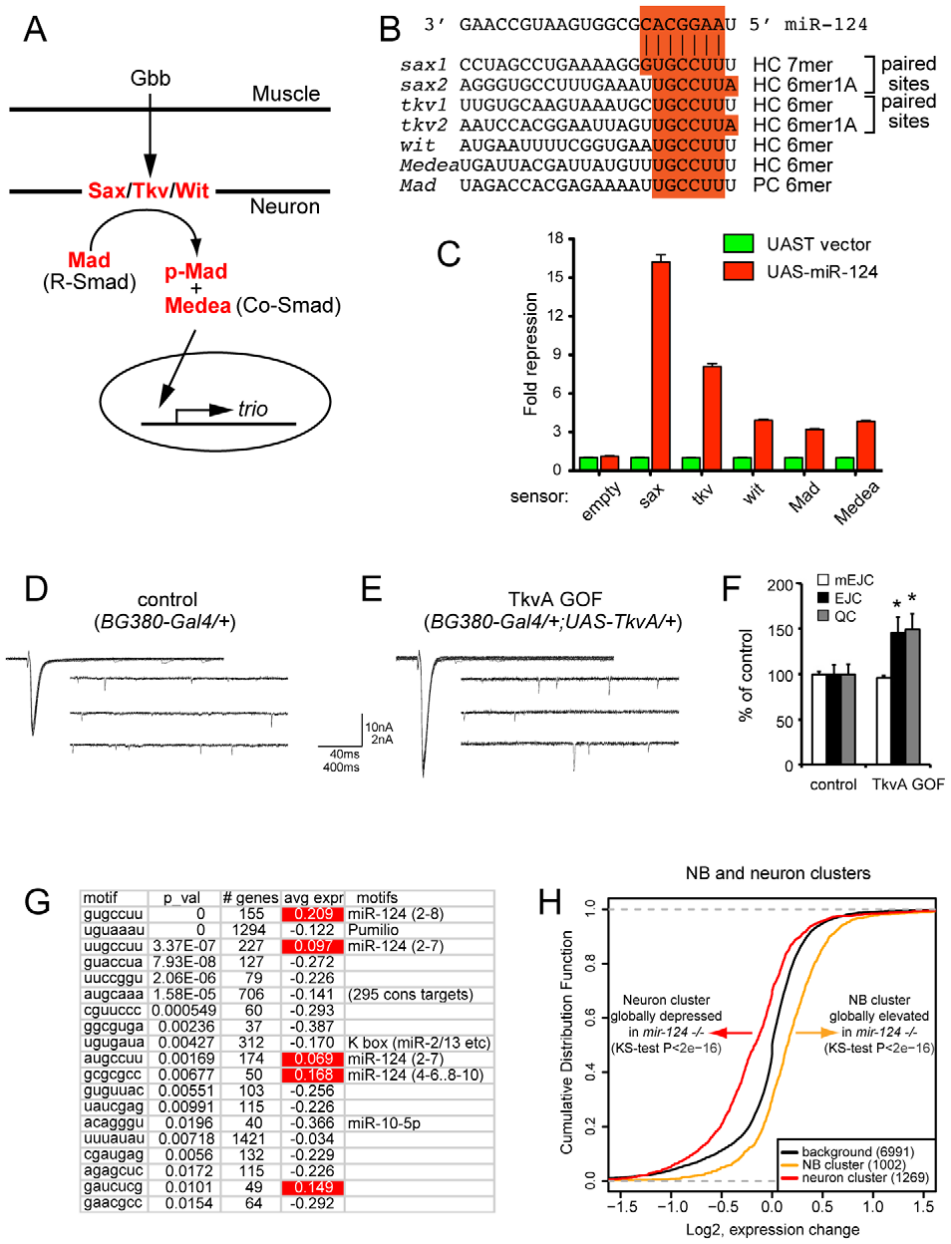
Functional interpretation of direct and indirect consequences of miR-124 loss. (A) Core components of the retrograde BMP signaling pathway at the NMJ. Release of the glass bottom boat (Gbb) ligand from the muscle activates BMP receptors (Sax, Tkv and Wit) in the neuron. Activated BMP receptors induce phosphorylation of Mad, which partners with Medea to activate target genes, such as *trio*. (B) miR-124 binding sites; HC = highly conserved and PC = poorly conserved (see also Figure S8). (C) Sensor assays in S2 cells confirm that the 3′ UTRs of all five BMP pathway components are responsive to miR-124. (D–F) Ectopic activation of Tkv receptor can phenocopy *mir-124* mutant electrophysiology. Representative traces of evoked (excitatory junction currents, EJC) and spontaneous (miniature EJC, mEJC) membrane currents recorded from muscle 6 in the third abdominal segment in *w[1118]; BG380-Gal4/+* (D) and *w[1118]; BG380-Gal4/+; UAS-TkvA/+* (E) wandering third-instar larvae. EJC contain 10 consecutive superimposed traces and mEJC are three traces of continuous recordings. (F) Quantification of mEJC, EJC, and quantal content (QC) for the indicated genotypes. Activated Tkv did not affect spontaneous activity, but caused significant increases in evoked currents and quantal content. n = 12 NMJs for each genotype. Error bars represent SEM, statistical tests by two-tailed t-test show *p<0.05. (G) miREDUCE analysis shows that variations of the miR-124 seed are strongly enriched amongst transcripts that increase in *mir-124* mutant cells (highlighted in red). Amongst motifs associated with decreased gene expression in *mir-124* mutants, the top motif corresponds to the Pumilio site; others motifs include the seeds of K box family miRNAs, miR-10-5p, and an orphan motif (AUGCAAA) with several hundred conserved matches (defined by TargetScan). (H) Cumulative distribution function (CDF) plots of gene in the neuron and NB clusters. The group of neural genes is shifted towards lower expression levels in the *mir-124* mutant, while the NB cluster is shifted towards higher expression.

Given that we failed to observe substantial contribution of mutual exclusion to the functional miR-124 target network, we sought connections between de-repressed miR-124 targets and mutant phenotypes. Amongst neural genes upregulated ∼2-fold in *mir-124* mutant cells and contain miR-124 binding sites in their 3′ UTRs were multiple members of the retrograde BMP signaling pathway, including the receptors *saxophone* (*sax*) (Figure 6C) and *wishful thinking* (*wit*), and the transcription factor *Mad* (Tables S1 and S2). Further inspection showed that another BMP receptor *thickveins* (*tkv*) and the co-Smad *Medea* also contain highly conserved miR-124 binding sites, although *tkv* mRNA was not upregulated in the microarray and *Medea* was not detected by this platform (even though it has a critical function in neurons). These five genes are core positive components of the retrograde BMP signaling pathway (Figure 8A), by which the target muscle activates BMP signaling in the neuron to control NMJ development and synaptic physiology [44].

Although many of these sites in BMP pathway targets were only 6mers (matching positions 2–7 of miR-124), all of them except the *Mad* site were well-conserved across Drosophilid evolution (Figure 8B and Figure S8), implying their functional constraint. Moreover, both *sax* and *tkv* contained closely paired sites that are predicted to function cooperatively [45]. We conducted sensor assays to examine the response of these targets to miR-124, and observed that all five targets were indeed repressed by ectopic miR-124, with especially strong repression of the *sax* and *tkv* sensors that contained conserved paired sites (Figure 8C). Since coordinate regulation of multiple aspects of an entire pathway by an individual miRNA is only rarely observed [1], [46], [47], this property is a distinctive aspect of the miR-124 target network.

Notably, we recently showed that misexpression of activated Sax and Tkv receptors in motoneurons increases synaptic activity without affecting NMJ structure [48], [49], similar to *mir-124* mutants. We conducted further experiments by expressing activated Tkv alone in motoneurons using *BG380-Gal4*. Activated Tkv did not affect spontaneous synaptic activity, as measured by miniature EJCs, but did increase both evoked EJCs and quantal content by 50% (Figure 8D–8F). These defects phenocopied the electrophysiological defects of *mir-124* mutant synapses (Figure 4E–4K). Although deregulation of other targets likely contributes to the observed *mir-124* mutant phenotypes, the similarity in electrophysiological defects upon deletion of miR-124 and overactivity of retrograde BMP signaling suggests that deregulation of this pathway may contribute to aberrant physiology of *mir-124* mutant synapses.

### Overactivity of other neural post-transcriptional regulators in *mir-124* mutants

The bioinformatic analyses presented thus far focused specifically on motifs of interest, e.g. miR-124 seeds. A complementary strategy is to assess what sequence motifs best explain global shifts in gene expression between control and experimental conditions. The miREDUCE algorithm performs an unbiased search for motifs that correlate with patterns of upregulated or downregulated expression changes [50]. Amongst 7-nt motifs associated with transcripts that increased in the *mir-124* mutant nervous system, the highest-scoring motif (*p*-value = 0) corresponded to the miR-124 seed region (positions 2–8), while the next highest-scoring motifs amongst globally upregulated transcripts corresponded to variations of 2–7 miR-124 seeds (Figure 8G). These encompassed larger gene cohorts than the canonical seed cohort (227 and 174, compared to 155 canonical seed targets), but were associated with more modest overall target over-accumulation, consistent with the directed CDF analysis (Figure 6G). The fourth-highest scoring motif (GCGCGCC) amongst up-regulated transcripts did not match a continuous region of miR-124, but exhibited notable similarity. It is not clear if such matching is biologically relevant, or a statistical anomaly related to its GC-rich character. In any case, these data provide clear evidence that the derepression of direct miR-124 targets is the major determinant causing gene upregulation in *mir-124* mutant cells.

The miREDUCE analysis also revealed several motifs associated with transcripts that were downregulated in the absence of miR-124. Two of these were seeds for K box miRNAs and for the Hox miRNA miR-10-5p (Figure 8G). Interestingly, we have earlier shown that a cluster of three K box miRNAs (*mir-2c*, *mir-13a* and *mir-13b-1*) is specifically expressed throughout the embryonic CNS [8], and other Hox miRNAs (e.g. *mir-iab-4* and *mir-iab-8*) are restricted to specific anterior-posterior domains in the CNS of germband-retracted embryos [51]. Therefore, the loss of the abundant CNS miRNA miR-124 may result in the overactivity of other CNS miRNAs.

Amongst motifs that did not match known miRNA seeds, we were struck by the enrichment of UGUAAAU amongst down-regulated transcripts, at a *p*-value = 0 (Figure 8G). This motif corresponds exactly to the Pumilio binding site [52]. *Drosophila* Pumilio was originally characterized as a critical translational repressor during embryonic patterning, but was later recognized to be re-expressed and regulate gene expression in neurons [53]–[55]. The FlyAtlas database confirmed high expression of *pumilio* in the larval central nervous system and adult head (http://www.flyatlas.org/). *Pum* transcript was only mildly upregulated in *mir-124* mutant cells, and available antibodies were not suitable for immunostaining (not shown). Nevertheless, the strong enrichment of Pumilio binding sites amongst transcripts downregulated in *mir-124* mutants suggests its overactivity. Interestingly, Pumilio is also known to regulate neuronal excitability [55], in addition to BMP signaling. Therefore, direct and indirect consequences may both contribute to electrophysiological defects caused by the absence of miR-124.

### Loss of miR-124 impairs neuroblast to neuronal transition

Having documented both primary and secondary effects of loss of miR-124 on neural gene expression, we asked whether such gene deregulation exerted a coherent overall effect on cell identity. Despite bioinformatic evidence for the mutual exclusion model (Figure 7A) [15], we do not find evidence for encroachment of epidermal characteristics within *mir-124* mutant neurons. Nevertheless, gene deregulation in *mir-124* mutant cells could be interpreted as a failure to consolidate the neural gene expression signature. Since *mir-124* is activated in neuroblasts and maintained in differentiated neurons (Figure 1), we hypothesized that the absence of miR-124 might be manifest in the transition from the neuroblast to neural state.

To study this, we took advantage of larval neuroblast and neuronal gene expression signatures defined by comparison of normal and various brain tumor mutants, which generate a high proportion of neuroblasts [56]. This yielded clusters of 1109 and 1415 unique genes that were mostly restricted to neuroblasts and neurons, respectively, of which 1002 and 1269 were expressed in miR-124+ cells. These gene lists overlapped rather poorly with direct miR-124 targets, and that the number of direct targets in the neuroblast and neuronal clusters was comparable (51 and 74, respectively). Therefore, miR-124 does not seem to have an overarching theme in, for example, directly targeting neuroblast genes. Nevertheless, we observed strikingly opposite behavior of neuroblast and neuronal genes as a whole, in the absence of *mir-124* (Figure 8H). Neuronal gene expression was globally decreased in miR-124:DsRed cells isolated from *mir-124* mutants compared to wild-type (p<2.2E-16). Reciprocally, we observed that neuroblast gene expression was globally increased in these mutant cells (p<2.2E-16). We infer from these gene expression patterns that the derepression of the miR-124 target network, impedes the normal transition of gene expression from neuroblasts to differentiated neurons in *mir-124* mutants. Altogether, our analyses reveal a complex set of primary and secondary effects on neuronal gene expression in *mir-124* mutants, which are collectively associated with behavioral dysfunction in larval and adult stages.

## Discussion

### Endogenous requirements for the highly conserved neural locus miR-124

Our studies of *Drosophila mir-124* demonstrate that its loss is compatible with grossly normal neural development and differentiation, despite broad changes in gene expression and global upregulation of direct miR-124 targets. Nevertheless, we detected many clear defects in these mutants, including short lifespan of adult males, defective larval locomotion, and aberrant synaptic transmission. The latter phenotype is perhaps reminiscent of reports that inhibition of *Aplysia* miR-124 similarly results in an increase in evoked EPSP amplitude [10]. We confirmed these phenotypes to be due to miR-124 loss, as shown by their rescue by a *mir-124* genomic transgene. Importantly, these phenotypes were obvious even under optimal culture conditions, demonstrating palpable requirements for this miRNA in the intact animal. It remains to be seen if synaptic overactivity in the *mir-124* mutant can be directly linked to the behavioral defects we observed at the organismal level (Figure 4). The electrophysiological defects in *mir-124* mutants phenocopy activation of BMP signaling at the synapse, and miR-124 directly targets multiple components of this pathway (Figure 8). Still, it remains possible that the many other gene expression changes in *mir-124* mutant neurons (Figure 6, Figure 7, Figure 8) contribute to its loss of function phenotype. Our detailed *in vivo* transcriptome-wide analysis of endogenous miR-124 targets sets the stage for future studies of how individual targets might affect different settings of miR-124 function.

Only a handful of other miRNA mutants are lethal or exhibit overt morphological defects [29], [57], suggesting that many miRNAs serve as robustness factors. For example, a *Drosophila mir-7* mutant exhibits minor cell specification defects, but these are enhanced by heat shock [58]. In addition, the introduction of many *C. elegans* “benign” miRNA mutants into genetically sensitized backgrounds uncovers a high frequency of phenotypes [59]. Interestingly, miR-124 is not required for normal dendrite formation *per se*, but its absence caused a broader distribution of dendrite numbers on ddaD and ddaE neurons, i.e. a “robustness” defect. We speculate that environmental or genetic stress may reveal additional requirements for miR-124 in development and differentiation of the nervous system.

In light of the broad roles ascribed to endogenous miR-124 in neurogenesis, neural differentiation, and neural physiology [60], all from antisense strategies, the extensive negative data from our *Drosophila mir-124* knockout are equally compelling. While we may not have examined the relevant neural subpopulation, our studies indicate that miR-124 is not required for gross aspects of neurogenesis and differentiation in the embryonic and larval nervous system. Similarly, *C. elegans* deleted for *mir-124*, which is expressed mostly in ciliated sensory neurons, do not reveal obvious defects in neural development [9]. Given that these invertebrate orthologs of miR-124 are identical in sequence to their vertebrate counterparts, and are highly and specifically expressed in their respective nervous systems, there is not strong reason *a priori* to suspect that miR-124 should not have comparable requirements amongst different animals. The analysis of vertebrate *mir-124* knockouts is therefore highly anticipated.

### The impact of endogenous *Drosophila* miR-124 on neuronal gene expression

The *Drosophila* system has been critical for elucidating fundamental features of miRNA target recognition in animals [3], [15], [46], [61]–[63], and for studying specific miRNA-target interactions that mediate phenotype [64]. However, it has been little-used to analyze the effects of miRNA-mediated gene regulation in the animal at the transcriptome-wide level. Perhaps the clearest example is the broad upregulation of maternal transcripts in early embryos lacking the *mir-309* cluster [65]. However, most miRNAs are tissue or cell-specific, and while it is much simpler to profile transcripts from whole flies, the inclusion of irrelevant cells can mask the action of the miRNA. For example, only 4/200 transcripts upregulated in *mir-8* mutant pupae appeared to be direct conserved targets [66].

By purifying cognate miRNA-expressing cells from wild-type and miRNA-mutant backgrounds, we were able to assess transcriptome-wide effects of genetic removal of miR-124 with precision. Our data provide a new perspective on the utilization of “anti-targeting” in *Drosophila*. Previously, miR-124 was selected as a particularly compelling case in which its *Drosophila* targets were depleted for *in situ* terms related to nervous system development, and enriched for terms related to epidermal development [15]. Since these tissues derive from a common developmental progenitor, the neuroectoderm, this led to a model in which miR-124 may solidify the neural fate by widespread suppression of epidermal genes that should be absent from neurons. We could confirm this bioinformatic correlation using an independently-derived set of miRNA targets (Figure 7A).

Nevertheless, two observations suggest that the feature of mutual exclusion in the *Drosophila* miR-124 network is of subtle consequence. First, derepressed target genes were not enriched for epidermally-expressed genes. This is consistent with the view that on the transcriptome-wide level, the exclusion of epidermal genes from miR-124-expressing cells is primarily enforced by transcriptional mechanisms. Second, miR-124 targets were preferentially amongst the higher-expressed transcripts in miR-124+ cells, even in wild-type. Moreover, as well-conserved targets were expressed at overall higher absolute levels than poorly-conserved targets in miR-124+ cells, we conclude that a dominant feature of the miR-124 target network has selected for substantial co-expression of the miRNA and its targets, perhaps to fine-tune their levels. This viewpoint is consistent with analyses of miR-124 targets in human [50], zebrafish [35] and *C. elegans* [9], indicating a unifying theme for this particular miRNA across animals.

Early manifestations of the miRNA world emerged from pervasive control of the *C. elegans* heterochronic pathway [67] and the *D. melanogaster* Notch pathway [1], [46] by miRNAs, and a few similar situations have been documented, i.e. direct targeting throughout the branched amino acid catabolism pathway by miR-277 [47] or repression of multiple components of fatty acid metabolism by miR-33 [68]. Nevertheless, it is rare for such dedicated target networks to be seen amongst the miRNA oeuvre. Amongst the broad network of miR-124 targets, we are struck by the coordinate targeting of multiple components of the retrograde BMP signaling pathway [44], including all three receptors (Sax/Tkv/Wit), the downstream transcription factor (Mad) and its cofactor (Medea). We recently showed that misexpression of activated Sax and Tkv receptors in motoneurons increases evoked excitatory junctional potentials without affecting spontaneous activity, very similar to that of *mir-124* mutants [48]. We extended this finding by analysis of activated Tkv alone (Figure 8D–8F). Therefore, deregulation of BMP signaling may contribute to the electrophysiological defects observed in *mir-124* mutants.

Still, a “one size fits all” description of miR-124 activity is not appropriate, since we certainly do observe a number of functional miR-124 targets whose predominant activities are in epidermal or other non-neural derivatives. Thus, the large miR-124 network accommodates a range of target properties [69], [70]. Derepression of a sufficient number of such non-neural transcripts may contribute collectively to the incomplete capacity of *mir-124* mutant cells to transition from a neuroblast to neuronal gene expression signature (Figure 8H).

### Cross-regulatory effects of *mir-124* loss on other modes of RNA–based regulation

One may speculate that dysfunction of miRNAs, which have large networks of targets, may trigger global changes in other modes of gene regulation. For example, overexpression of individual miRNAs or siRNAs can de-repress endogenous regulation via non-cognate miRNAs, possibly reflecting a titration mechanism [71]. In addition to a global effect on neuroblast-to-neural transition, we observed that genes downregulated upon *in vivo* loss of miR-124 were enriched for seeds of K box miRNAs and miR-10-5p (Figure 8G). This is potentially consistent with a model in which absence of this abundant miRNA frees up AGO1 complexes to accept other neural miRNAs, yielding their overactivity. Another plausible mechanism might be that miR-124 represses a transcriptional repressor of these other miRNAs.

We also observed that Pumilio binding sites were strongly associated with downregulated transcripts in *mir-124* mutants. Pumilio is well-characterized as a neural RNA binding protein and translational regulator, and affects synaptic function and dendrite morphogenesis [53]–[55], which we also observed to be miR-124-regulated settings. Predictions of conserved miRNA binding sites (e.g. TargetScan or mirSVR) did not identify miR-124 target sites in the annotated *pumilio* 3′ UTR or CDS; however modENCODE data [72] revealed that *pumilio* transcription extends >2 kb downstream of its annotated 3′ end. The regulatory potential of such long *pumilio* 3′ UTR isoforms remains to be studied. Other possibilities are that miR-124 regulates a transcriptional regulator of *pumilio*, or that Pumilio activity is altered in *mir-124* mutants. Future studies should address the cross-talk of post-transcriptional regulation in neurons mediated by miR-124, neuronal miRNAs and Pumilio.

## Materials and Methods

### *Drosophila* stocks

Deletion alleles of *mir-124* were generated using ends-out recombination [73]. ∼4 kb left and right homology arms were amplified using PCR (Table S3 for primer sequences) and cloned into pW25.2 donor targeting vector, and injected into *w[1118]* (BestGene, Chino Hills CA). Donor insertions on chromosome X or III were used for *mir-124* targeting, and were crossed to flies carrying heat shock-inducible FLP recombinase and I-SceI endonuclease, to mobilize the miRNA targeting element from the donor chromosome and linearize the excised fragment. Adult flies collected from larvae subjected to 1 hr heat shock at 37°C were crossed with balancer flies that contain second and third chromosome markers that allow mapping of *mini-white*. For flies in which *mini-white* mapped to chromosome II, PCR was performed to verify the integration of the targeting construct at the *mir-124* locus using primers that bind outside the left homology arm and within unique vector sequence downstream of the left homology arm but upstream of the *mini-white* gene. Only flies with correct targeting produce a ∼4.5 kb PCR fragment. Excision of the *mini-white* gene using *hs-Cre* recombinase was verified by PCR generating a diagnostic ∼500 bp fragment. Primer sets for *mir-124* validation are listed in the Table S3.

The *mir-124* rescue transgene was generated by injection of P[acman] clone CH322-39N16 into attP16 strain [74] (Genetic Services Inc., MA). *miR-124:dsRed* was generated by cloning 4 kb upstream of the hairpin into Red-H-Stinger [75]. ∼400 bp genomic fragment containing the *pre-mir-124* sequence was cloned into the UAS-dsRed [47] to generate the *UAS-dsRed-mir-124* transgene. To analyze larval neuroblast clones, we heat-shocked *hsflp, tubGal4, UAS-GFP; FRT40A, tubGal80/FRT40A mir-124[6]* for 37°C for 90 minutes at 24 hr ALH (after larval hatching) and dissected at 96 hr ALH.

### In situ hybridization and immunohistochemistry

DNA templates were generated by PCR amplification of ∼1 kb genomic sequences containing the miRNA hairpin; T7 promoter was attached to the antisense strand primers. See Table S3 for primer sequences. Antisense digoxigenin-labeled RNA probes were generated by in vitro transcription with the DNA template and T7 polymerase according to the standard protocol (Roche). Embryos were fixed and prepared as described previously [8].

For immunostaining, embryos were dechorionated in bleach and fixed in 4% formaldehyde for 20 min followed by devitellination. Fixed embryos were stored in −20°C at least one overnight before staining. Embryos were rehydrated in 50% methanol, washed in PBSTw (0.1% Tween-20 in PBS) and then blocked in 0.5% PBSBT (0.5% BSA and 0.1% Triton X-100 in PBS). Both primary and secondary antibodies were incubated overnight in 4°C. The following primary antibodies were used: rabbit-anti-dsRed (1∶500, Clontech), rat-anti-Elav (1∶250, DSHB), rat-anti-deadpan (1∶50, Doe lab), rabbit-anti-Hunchback (1∶200, Doe lab), guinea pig anti-Miranda (1∶500, Doe lab), mouse-anti-Prospero (1∶20, DSHB), mouse-anti-Eve (1∶5, DSHB), mouse-anti-Repo (1∶20, DSHB), mouse-anti-22C10 (1∶100, DSHB). Alexa Fluor-488, 568, 647 secondary antibodies were from Molecular Probes and used at 1∶500. For staining of larval neuromuscular junction, 3^rd^ instar wandering larvae were dissected as described [76]. Alexa Fluor-568 phalloidin (1∶400, Invitrogen) and FITC-HRP (1∶250, Jackson ImmunoResearch) were used to visualize the F-actin and NMJ. Images were captured with a Leica TCS confocal microscope. Synaptic boutons and NMJ expansion were quantified with the Leica software.

### Behavioral assays

Lethal phase analysis. Flies were cultured in 25°C and allowed to lay eggs for 12 hours. For each genotype, 100 embryos were collected and transferred to an apple juice plate and each plate was scored for the number of hatched larvae and pupae. The number of eclosed adults was scored everyday from day 8 to 13 for each genotype. Experiments were repeated five times.

Life span assay. Male flies were collected within 24 hr of eclosion and maintained in 29°C in low density (5 males/vial, 20 vials per genotype). Flies were transferred to fresh vials every 2∼3 days and scored for survivors across the timecourse.

Larval locomotion assay. Larval locomotion was assayed as described [77] but without odor source. Briefly, single mid-3^rd^ instar larva was placed on a 96 well plate lid covered with 3% agarose and animal locomotion was recorded by a CCD camera for 1 min since its first movement. Data was collected and analyzed with the Ethovision software (Noldus). 15∼30 animals were tested for each genotype.

### Dendrite analysis

*Gal4^221^* driver was used to label ddaD and ddaE neurons with mCD8-GFP and drive the expression of transgenes. The dendritic morphology of GFP-labeled dorsal sensory neurons was recorded by confocal (Nikon, D-Eclipse C1). One ddaD neuron and one ddaE neuron were recorded from A3 segment of each larva and their dendrites were counted as described [78]. Briefly, dendritic ends of ddaD or ddaE neurons were identified visually and highlighted with dots, which were counted using Adobe Photoshop. The data were analyzed by the Wilcoxon test and F test.

### Electrophysiology

Wandering third instar larvae were dissected in cold HL3 solution without Ca^2+^ following standard protocol [79], using the *mir-124* genotypes described above and *BG380-Gal4*>*UAS-TkvA* [80]. The spontaneous (mEJC) and evoked (EJC) membrane currents were recorded from muscle 6 in abdominal segment A3 with standard two-electrode voltage-clamp technique [80]. All the recordings were performed at room temperature in HL3 solution containing 0.5 mM Ca^2+^. The current recordings were collected with AxoClamp2B amplifier (Molecular Devices Inc.) and stored on a desk top computer using Clampex 9.2 software (Molecular Devices Inc.). The nerve stimulation was delivered through a suction electrode, which held the cut nerve bundle. In all voltage clamp recordings, muscles were held at −80 mV. The holding current was less than5 nA for 90% of the recordings and we rejected any recording that required more than 10 nA current to maintain the holding potential. The amplitudes of mEJC and EJC were measured using Mini Analysis 6.0.3 software (Synaptosoft) and verified by eye. QC was calculated by dividing the mean EJC amplitude by mean mEJC amplitude. The recording traces were generated with Origin 7.5 software (Origin Lab).

Data are presented as Mean ± SEM (*n* = number of NMJs unless otherwise indicated). Histograms were generated using Excel software (Microsoft Corporation). Statistical significance was determined using PASW 7.0 software (SPSS Inc.). Each data set was first subjected to a variance test. In the absence of a significant difference, One-way ANOVA followed by Tukey post-hoc test was applied. If there were differences in variance, Games-Howell post-hoc test was applied.

### FACS and microarray analysis

Wild type (*mir-124:dsRed*) and mutant (*mir-124*[*del12/12*]; *mir-124:dsRed*) flies were raised in collection cages at 25°C. 10∼16 hours embryos were collected and dechorionated in house bleach solution for 2∼3 min. Then embryos were washed in 80% ethanol for 5 min with occasional vortex and rinsed in modified Schneider media supplemented with 2% FBS, 0.1% Pen/Strep and 0.005 mg/ml Gentamicin for 3 times. Embryos were transferred to supplemented Schneider media (20% FBS) and homogenized in a 7 ml tissue grinder (Wheaton #357542) until no large clumps were visible. Homogenate was transferred to an eppendorf tube and spun at 5000 rpm for 5 min. Pellets were resuspended in 0.01% trypsin in unsupplemented Schneider media (without FBS) and incubated for 5 min. Dissociated cells were purified by passage through a cell strainer cap (BD Falcon #352235) twice and finally resuspended in 20% FBS supplemented Schneider media.

Fluorescence activated cell sorting was carried out immediately after preparation using a MoFlo flow cytometer (Cytomation) in the MSKCC Flow Cytometry Core Facility. Total RNA from the sorted cells was extracted using Trizol LS (Invitrogen). To enhance precipitation, RNA was precipitated with glycogen (Ambion). RNA samples including 3 biological replicates for each genotype were labeled and hybridized to the GeneChip *Drosophila* Genome 2.0 Array (Affymetrix) by the MSKCC Genomics Core Laboratory. Primers for qPCR validation of *Dsred* and *pri-mir-124* are listed in Table S3.

### Sensor assays and qPCR

3′ UTRs of predicted miR-124 targets were cloned into the psiCHECK-2 vector (Promega) using cold fusion cloning (System Biosciences). Sensor plasmid and *ub-Gal4* were cotransfected with *UAS-DsRed-miR-124* or empty *pUAST* vector into S2-R+ cells using Effectene (Qiagen). Luciferase activities were measured by Dual-Glo Luciferase assay (Promega). To verify several gene expression changes in microarray, qRT-PCR were performed using SYBR Green reagent (Applied Biosystems) and the CFX96 Real-Time PCR detection system (Bio-Rad). Primers used for cloning 3′ UTR sensors and performing qPCR are listed in Table S3.

### Computational analysis

Microarray data were normalized using the GCRMA bioconductor package and log enrichment values were computed using the limma package with p-values adjusted for multiple hypothesis using FDR. For genes with multiple probes, the probe with lowest adjusted p-value was selected.

Targets were predicted and scored using miRanda-mirSVR method [41]. Predicted target sites were restricted to include perfect seed complementarity (positions 2–7) and non-canonical sites with favorable mirSVR scores (<−0.1). Empirical cumulative distributions were computed using the R ecdf function on mutually exclusive gene sets and P-values were computed by the Kolmogorov-Smirnov non-parametric test. Detection of sequence motifs that are correlated with log-fold expression changes was performed using miReduce [50] with motif length parameter of 7 and p-value cutoff< = 0.05.

The predicted miR-124 target sites were partitioned into well-conserved and poorly-conserved based on the Multiz 15 fly species alignment in the UCSC genome browser [42]. Target sequences where at least 5 sequences (including *D. pseudoobscura*) from *D. sechellia*, *D. simulans*, *D. yakuba*, *D. erecta*, *D. ananassae* and *D. pseudoobscura* were identical to *D. melanogaster* were considered well-conserved, all other sequences were labeled poorly-conserved.

Enrichment of Gene Ontology annotations and in-situ gene expression profiles [43] were computed with Fisher's exact test, using the Bonferroni correction for multiple hypothesis testing. Up- and down regulated genes were required to have fold change >30% and p-value<0.05.

## Supporting Information

Figure S1 Expression of the miR-124:dsRed reporter in stage 8 embryos. Although the level of DsRed is quite low, over-exposing makes it evident that the pattern overlaps well with the pan-neuroblast marker Deadpan. Boxed region in the merge panel highlights asymmetric segregation of Prospero into the ganglion mother cell. In situ hybridization for *pri-mir-124* confirms detection of nuclear primary transcripts at stage 8 (arrows, inset). Detection of these initial nascent transcripts required overstaining, resulting in a general deposition of chromophore.
(PDF)

Figure S2 Lethal phase analysis of *mir-124* mutants. Substantial embryonic lethality was observed in *mir-124* mutants, which was demonstrably (although not completely) rescued by the *mir-124* genomic transgene. No differences in larval or pupal survival were seen.
(PDF)

Figure S3 Analysis of CNS markers in *mir-124* mutants. Representative images of Even-skipped (A,B) and Hunchback (C,D) expression in the neuronal layers, and Miranda (E, F) and Deadpan (G, H) expression in the neuroblast layers of wild type and *mir-124[6/6]* mutants are shown. Mira, Dpn, and Eve wild type images are taken from *mir-124* genomic rescue embryos, and the Hb image is taken from *yw* embryo. Each panel is a maximal z-projection through the ventral nerve cord at stage 16, showing hemisegments T2 through A4. Anterior is to the left. White dotted line indicates midline. Scale bar = 20 µm.
(PDF)

Figure S4 Loss of *mir-124* does not result in abnormal axonal architecture as labeled by 22C10 in st15 embryos, either in CNS (A,B) or PNS (C,D). A, B are ventral views comprising 5–6 segments; anterior is to the top. We focused on a restricted z-series to highlight CNS architecture; therefore the PNS is not well-visualized in these images. C,D are lateral views of entire embryos to highlight the PNS; anterior is to the left.
(PDF)

Figure S5 Expression of the miR-124:dsRed reporter in larval CNS. (A–A′″) Colabeling of miR-124:dsRed reporter in larval CNS with Elav (marking neurons) and NC82 (marking neuropile) in a single confocal slice. miR-124:dsRed is active in the brain and the ventral nerve cord (VNC). (B–B′″) Colabeling of the miR-124:dsRed in the brain with Deadpan (marking neuroblasts) and Elav. miR-124:dsRed is mostly active in the central complex, both in neuroblasts and neurons; it is expressed only weakly in the optic lobe. Shown is an overlay of dorsal brain z-sections. (C–C′″) MARCM analysis in control larval brain clones to mark the lineages produced by single neuroblasts. GFP+ clones maintain a single neuroblast (marked by large Dpn+ cells in red, arrows in C and C′) and can generate multiple neurons (as marked by Elav in blue). Note that this is a relatively superficial cross-section and additional GFP-labeled neurons in the clones are located in deeper layers. OL, optic lobe; CC, central complex.
(PDF)

Figure S6 Quantitative analysis of locomotion defects in *mir-124* mutant larvae. 15–30 larvae of the indicated genotypes were tracked for one minute each. The total distance traveled was quantified. *mir-124* exhibited less movement, and their behavior was restored by inclusion of a 19 kb *mir-124* rescue transgene. ***p<0.001.
(PDF)

Figure S7 Absolute expression of miR-124 target genes in miR-124:DsRed+ cells. Left panel is the same as in main Figure 7B, indicating that both miR-124 well-conserved and poorly-conserved targets are expressed at relatively high levels in both wt and *mir-124* mutants.
(PDF)

Figure S8 Conservation of miR-124 target sites amongst components of the retrograde BMP signaling pathway. Left is the retrograde BMP signaling pathway, red are miR-124 targets, which are all on the positive direction of BMP signaling. hiw, ema and spict are negative regulators of BMP signaling, loss of which leads to NMJ overgrowth. Below are the miR-124 targeting of BMP pathway genes, red boxes on the conservation graphs indicate sequences pairing with miR-124 seed region and their extent of conservation. We consider target sites to be highly conserved if they are preserved outside of melanogaster group species (*D. pseudoobscura*, *D. persimilis*, *D. willistoni*, *D. mojavensis*, *D. virilis* and/or *D. grimshawi*). Only the Mad miR-124 site is restricted to melanogaster group species, but it is perfectly conserved amongst these five genomes.
(PDF)

Table S1 Gene expression changes in *mir-124* mutants. The first worksheet summarizes gene expression between miR-124:DsRed+ cells isolated from wild-type and *mir-124−/−* 10-16 hr embryos. The second worksheet summarizes the top upregulated genes in *mir-124* mutant cells. About half of these contain miR-124 target sites. Note that the top-upregulated gene, white, is a consequence of genetic background, since the *mir-124* knockout is marked by an extra copy of mini-white. The third worksheet summarizes expression of genes present in *mir-124−/−* that were not called present in wild-type. Few of these contain miR-124 target sites, and all of these are lowly-expressed at best, suggesting that these differences do not contribute substantially to direct changes in gene expression in *mir-124* mutants.
(XLSX)

Table S2 GO term enrichments in *mir-124* expression data. Based on the data in Table S1, this series of worksheets summarizes GO term enrichments found in “all” up- and down-regulated genes as well as in just upregulated genes bearing miR-124 target sites; the latter were also separated on the basis of miR-124 site conservation.
(XLSX)

Table S3 Primer sequences used for cloning and gene expression analysis.
(XLSX)
