# Supplementary material for: Neurophysiological Defects and Neuronal Gene Deregulation in Drosophila mir-124 Mutants
Source: PLoS Genet. 2012 Feb 9;8(2):e1002515. doi: 10.1371/journal.pgen.1002515 (PMC3276548; doi:10.1371/journal.pgen.1002515)
Supplement: Figure S1 — Expression of the miR-124:dsRed reporter in stage 8 embryos. Although the level of DsRed is quite low, over-exposing makes it evident that the pattern overlaps well with the pan-neuroblast marker Deadpan. Boxed region in the merge panel highlights asymmetric segregation of Prospero into the ganglion mother cell. In situ hybridization for pri-mir-124 confirms detection of nuclear primary transcripts at stage 8 (arrows, inset). Detection of these initial nascent transcripts required overstaining, resulting in a general deposition of chromophore. (PDF) [file pgen.1002515.s001.pdf]

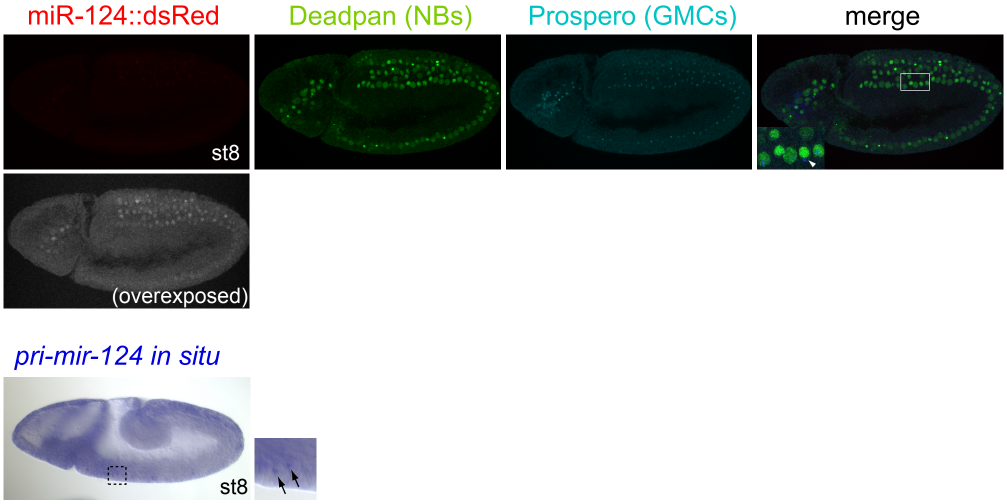

Supplementary Figure 1. Expression of the miR-124:dsRed reporter in stage 8 embryos. Although the level of DsRed is quite low, over-exposing makes it evident that the pattern overlaps well with the pan-neuroblast marker Deadpan. Boxed region in the merge panel highlights asymmetric segregation of Prospero into the ganglion mother cell. In situ hybridization for pri-mir-124 confirms detection of nuclear primary transcripts at stage 8 (arrows, inset). Detection of these initial nascent transcripts required overstaining, resulting in a general deposition of chromophore.

Supplementary Figure 1
Sun et al
