# Supplementary material for: Neurophysiological Defects and Neuronal Gene Deregulation in Drosophila mir-124 Mutants
Source: PLoS Genet. 2012 Feb 9;8(2):e1002515. doi: 10.1371/journal.pgen.1002515 (PMC3276548; doi:10.1371/journal.pgen.1002515)
Supplement: Figure S2 — Lethal phase analysis of mir-124 mutants. Substantial embryonic lethality was observed in mir-124 mutants, which was demonstrably (although not completely) rescued by the mir-124 genomic transgene. No differences in larval or pupal survival were seen. (PDF) [file pgen.1002515.s002.pdf]

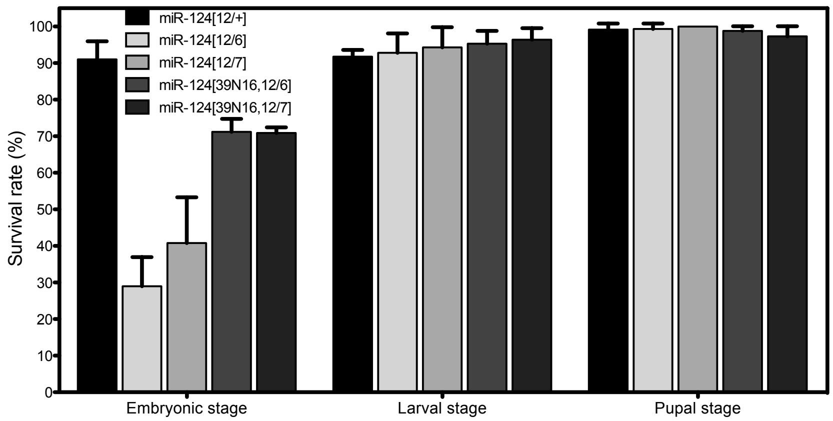

Supplementary Figure 2. Lethal phase analysis of *mir-124* mutants. Substantial embryonic lethality was observed in *mir-124* mutants, which was demonstrably (although not completely) rescued by the *mir-124* genomic transgene. No differences in larval or pupal survival were seen.

Supplementary Figure 2
Sun et al
