# Supplementary material for: Neurophysiological Defects and Neuronal Gene Deregulation in Drosophila mir-124 Mutants
Source: PLoS Genet. 2012 Feb 9;8(2):e1002515. doi: 10.1371/journal.pgen.1002515 (PMC3276548; doi:10.1371/journal.pgen.1002515)
Supplement: Figure S3 — Analysis of CNS markers in mir-124 mutants. Representative images of Even-skipped (A,B) and Hunchback (C,D) expression in the neuronal layers, and Miranda (E, F) and Deadpan (G, H) expression in the neuroblast layers of wild type and mir-124[6/6] mutants are shown. Mira, Dpn, and Eve wild type images are taken from mir-124 genomic rescue embryos, and the Hb image is taken from yw embryo. Each panel is a maximal z-projection through the ventral nerve cord at stage 16, showing hemisegments T2 through A4. Anterior is to the left. White dotted line indicates midline. Scale bar = 20 µm. (PDF) [file pgen.1002515.s003.pdf]

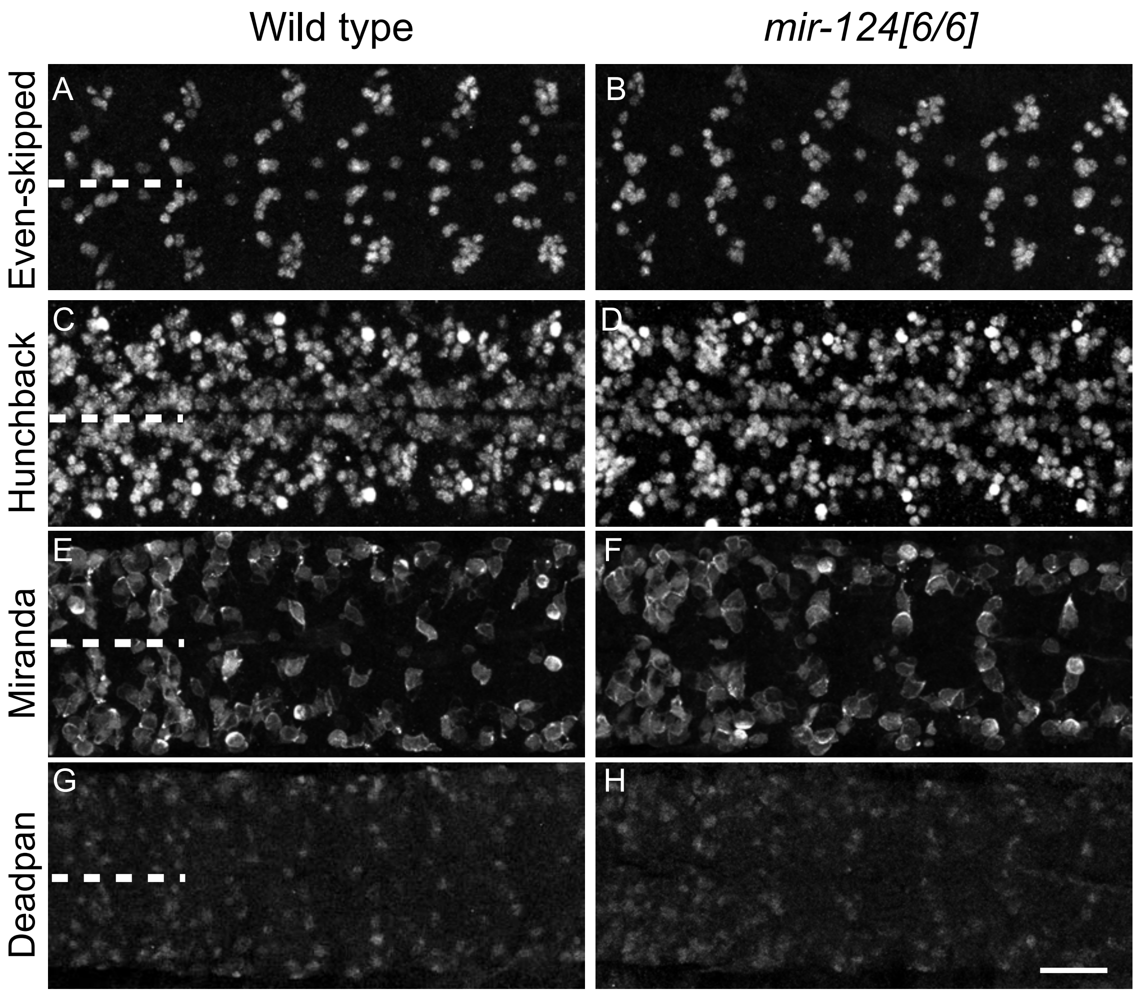

Supplementary Figure 3. Analysis of CNS markers in *mir-124* mutants.
Representative images of Even-skipped (A,B) and Hunchback (C,D) expression in the neuronal layers, and Miranda (E, F) and Deadpan (G, H) expression in the neuroblast layers of wild type and *mir-124[6/6]* mutants are shown. Mira, Dpn, and Eve wild type images are taken from *mir-124* genomic rescue embryos, and the Hb image is taken from yw embryo. Each panel is a maximal z-projection through the ventral nerve cord at stage 16, showing hemisegments T2 through A4. Anterior is to the left. White dotted line indicates midline. Scale bar = 20μm

Supplementary Figure 3
Sun et al
