# Supplementary material for: Neurophysiological Defects and Neuronal Gene Deregulation in Drosophila mir-124 Mutants
Source: PLoS Genet. 2012 Feb 9;8(2):e1002515. doi: 10.1371/journal.pgen.1002515 (PMC3276548; doi:10.1371/journal.pgen.1002515)
Supplement: Figure S4 — Loss of mir-124 does not result in abnormal axonal architecture as labeled by 22C10 in st15 embryos, either in CNS (A,B) or PNS (C,D). A, B are ventral views comprising 5–6 segments; anterior is to the top. We focused on a restricted z-series to highlight CNS architecture; therefore the PNS is not well-visualized in these images. C,D are lateral views of entire embryos to highlight the PNS; anterior is to the left. (PDF) [file pgen.1002515.s004.pdf]

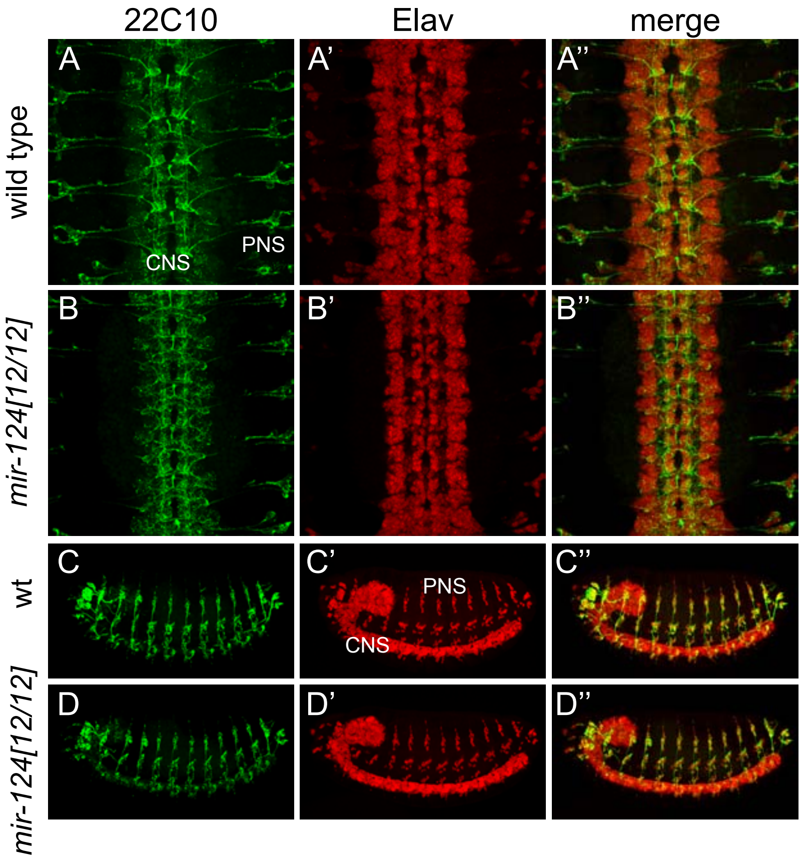

Supplementary Figure 4. Loss of *mir-124* does not result in abnormal axonal architecture as labeled by 22C10 in st15 embryos, either in CNS (A,B) or PNS (C,D). A, B are ventral views comprising 5-6 segments; anterior is to the top. We focused on a restricted z-series to highlight CNS architecture; therefore the PNS is not well-visualized in these images. C,D are lateral views of entire embryos to highlight the PNS; anterior is to the left.

Supplementary Figure 4
Sun et al
