# Supplementary material for: Neurophysiological Defects and Neuronal Gene Deregulation in Drosophila mir-124 Mutants
Source: PLoS Genet. 2012 Feb 9;8(2):e1002515. doi: 10.1371/journal.pgen.1002515 (PMC3276548; doi:10.1371/journal.pgen.1002515)
Supplement: Figure S5 — Expression of the miR-124:dsRed reporter in larval CNS. (A–A′″) Colabeling of miR-124:dsRed reporter in larval CNS with Elav (marking neurons) and NC82 (marking neuropile) in a single confocal slice. miR-124:dsRed is active in the brain and the ventral nerve cord (VNC). (B–B′″) Colabeling of the miR-124:dsRed in the brain with Deadpan (marking neuroblasts) and Elav. miR-124:dsRed is mostly active in the central complex, both in neuroblasts and neurons; it is expressed only weakly in the optic lobe. Shown is an overlay of dorsal brain z-sections. (C–C′″) MARCM analysis in control larval brain clones to mark the lineages produced by single neuroblasts. GFP+ clones maintain a single neuroblast (marked by large Dpn+ cells in red, arrows in C and C′) and can generate multiple neurons (as marked by Elav in blue). Note that this is a relatively superficial cross-section and additional GFP-labeled neurons in the clones are located in deeper layers. OL, optic lobe; CC, central complex. (PDF) [file pgen.1002515.s005.pdf]

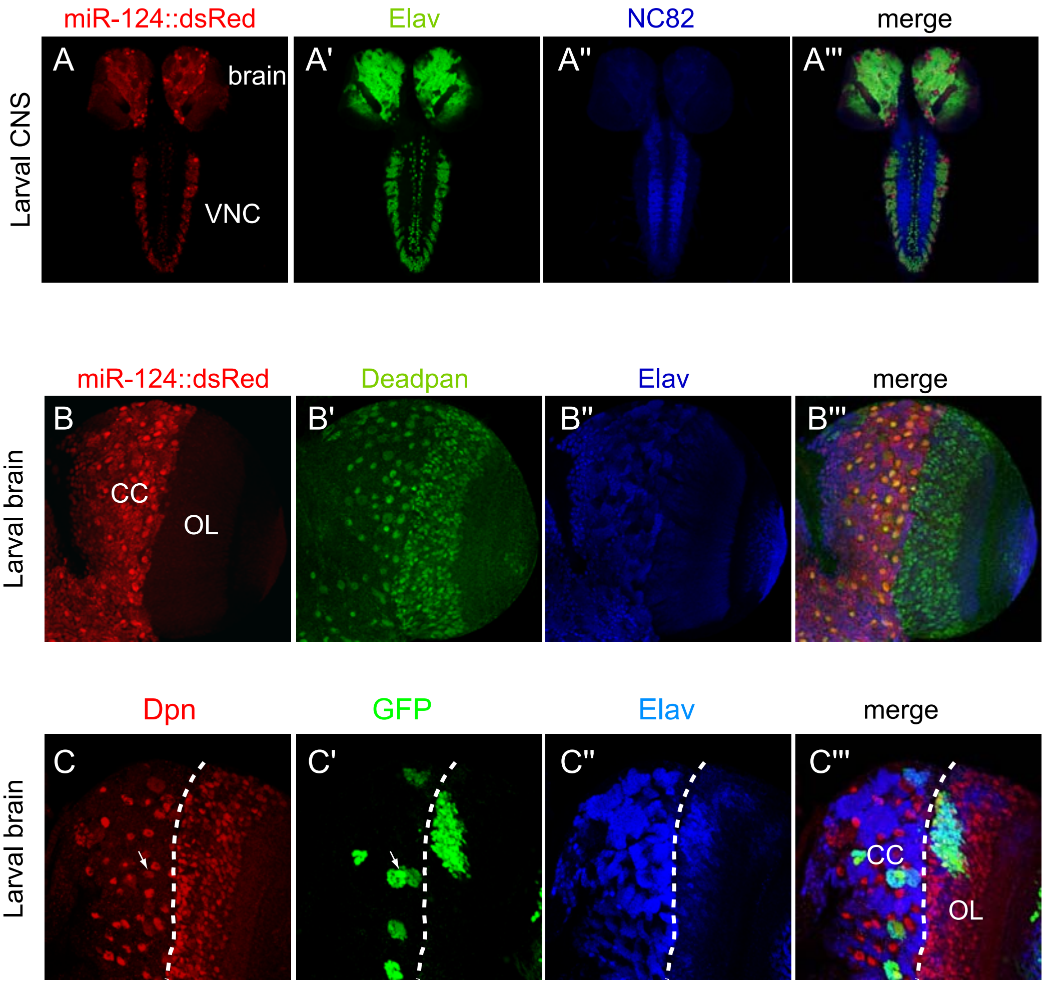

Supplementary Figure 5. Expression of the miR-124:dsRed reporter in larval CNS.
(A-A''') Colabeling of miR-124:dsRed reporter in larval CNS with Elav (marking neurons) and NC82 (marking neuropile) in a single confocal slice. miR-124:dsRed is active in the brain and the ventral nerve cord (VNC).

(B-B''') Colabeling of the miR-124:dsRed in the brain with Deadpan (marking neuroblasts) and Elav. miR-124:dsRed is mostly active in the central complex, both in neuroblasts and neurons; it is expressed only weakly in the optic lobe. Shown is an overlay of dorsal brain z-sections.

(C-C''') MARCM analysis in control larval brain clones to mark the lineages produced by single neuroblasts. GFP+ clones maintain a single neuroblast (marked by large Dpn+ cells in red, arrows in C and C') and can generate multiple neurons (as marked by Elav in blue). Note that this is a relatively superficial cross-section and additional GFP-labeled neurons in the clones are located in deeper layers.
OL, optic lobe; CC, central complex.
