# Supplementary material for: Neurophysiological Defects and Neuronal Gene Deregulation in Drosophila mir-124 Mutants
Source: PLoS Genet. 2012 Feb 9;8(2):e1002515. doi: 10.1371/journal.pgen.1002515 (PMC3276548; doi:10.1371/journal.pgen.1002515)
Supplement: Figure S6 — Quantitative analysis of locomotion defects in mir-124 mutant larvae. 15–30 larvae of the indicated genotypes were tracked for one minute each. The total distance traveled was quantified. mir-124 exhibited less movement, and their behavior was restored by inclusion of a 19 kb mir-124 rescue transgene. ***p<0.001. (PDF) [file pgen.1002515.s006.pdf]

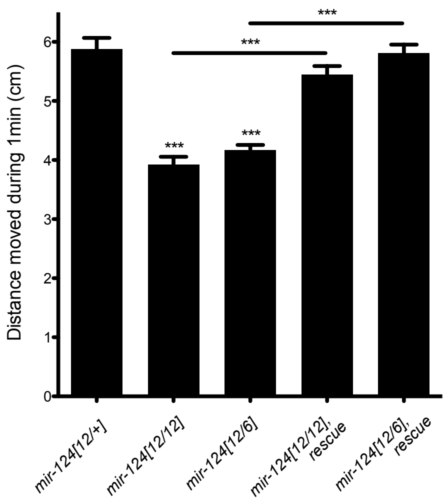

Supplementary Figure 6. Quantitative analysis of locomotion defects in *mir-124* mutant larvae. 15-30 larvae of the indicated genotypes were tracked for one minute each. The total distance traveled was quantified. *mir-124* mutants exhibited less movement, and their behavior was restored by inclusion of a 19kb *mir-124* rescue transgene. ***p<0.001.

Supplementary Figure 6
Sun et al
