# Supplementary material for: Neurophysiological Defects and Neuronal Gene Deregulation in Drosophila mir-124 Mutants
Source: PLoS Genet. 2012 Feb 9;8(2):e1002515. doi: 10.1371/journal.pgen.1002515 (PMC3276548; doi:10.1371/journal.pgen.1002515)
Supplement: Figure S7 — Absolute expression of miR-124 target genes in miR-124:DsRed+ cells. Left panel is the same as in main Figure 7B, indicating that both miR-124 well-conserved and poorly-conserved targets are expressed at relatively high levels in both wt and mir-124 mutants. (PDF) [file pgen.1002515.s007.pdf]

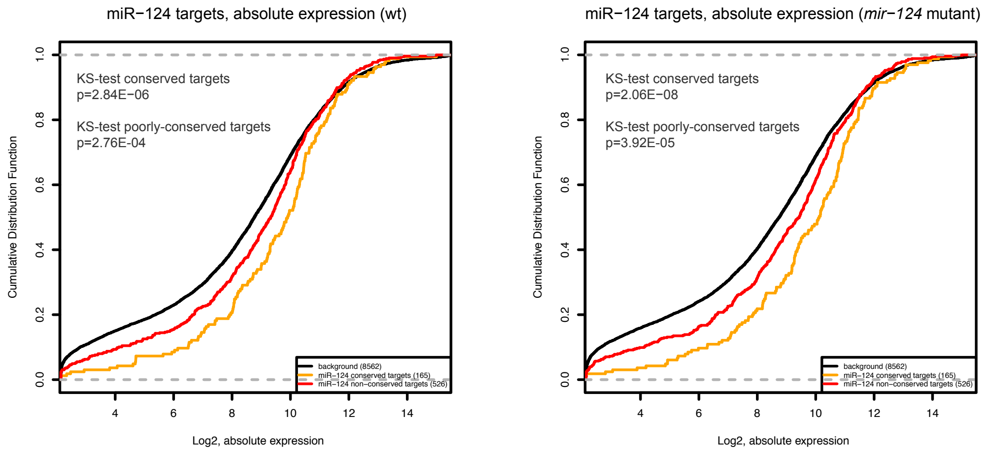

Supplementary Figure 7. Absolute expression of miR-124 target genes in miR-124:DsRed+ cells. Left panel is the same as in main Figure 7B, indicating that both miR-124 well-conserved and poorly-conserved targets are expressed at relatively high levels in both wt and *mir-124* mutants.

Supplementary Figure 7
Sun et al
