# Supplementary material for: Neurophysiological Defects and Neuronal Gene Deregulation in Drosophila mir-124 Mutants
Source: PLoS Genet. 2012 Feb 9;8(2):e1002515. doi: 10.1371/journal.pgen.1002515 (PMC3276548; doi:10.1371/journal.pgen.1002515)

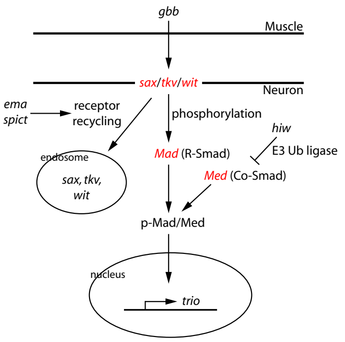

Supplementary Figure 8. Conservation of miR-124 target sites amongst components of the retrograde BMP signaling pathway.

Left is the retrograde BMP signaling pathway, red are miR-124 targets, which are all on the positive direction of BMP signaling. hiw, ema and spict are negative regulators of BMP signaling, loss of which leads to NMJ overgrowth.

Below are the miR-124 targeting of BMP pathway genes, red boxes on the conservation graphs indicate sequences pairing with miR-124 seed region and their extent of conservation.
We consider target sites to be highly conserved if they are preserved outside of melanogaster group species (D. pseudoobscura, D. persimilis, D. willistoni, D. mojavensis, D. virilis and/or D. grimshawi).
Only the Mad miR-124 site is restricted to melanogaster group species, but it is perfectly conserved amongst these five genomes.

Retrograde BMP receptors (sax, tkv, wit)

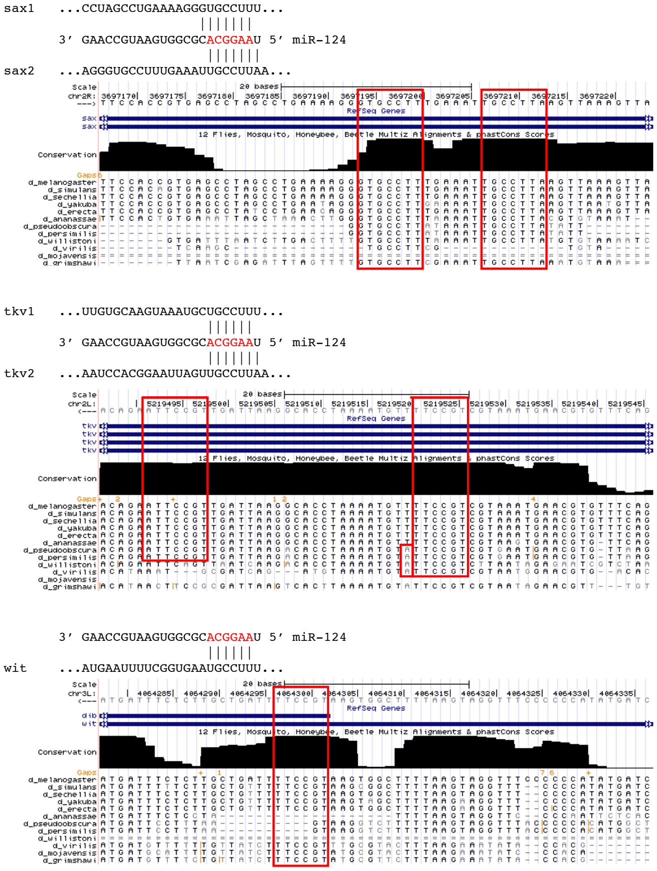

BMP transcription factors (Mad, Medea)

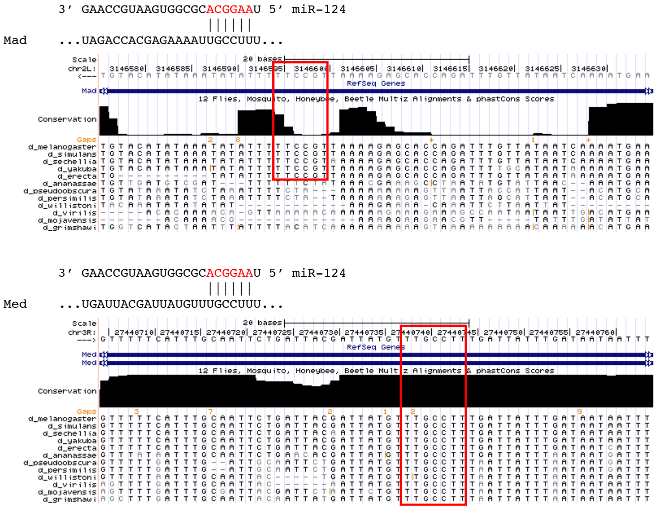

Supplement: Figure S8 — Conservation of miR-124 target sites amongst components of the retrograde BMP signaling pathway. Left is the retrograde BMP signaling pathway, red are miR-124 targets, which are all on the positive direction of BMP signaling. hiw, ema and spict are negative regulators of BMP signaling, loss of which leads to NMJ overgrowth. Below are the miR-124 targeting of BMP pathway genes, red boxes on the conservation graphs indicate sequences pairing with miR-124 seed region and their extent of conservation. We consider target sites to be highly conserved if they are preserved outside of melanogaster group species (D. pseudoobscura, D. persimilis, D. willistoni, D. mojavensis, D. virilis and/or D. grimshawi). Only the Mad miR-124 site is restricted to melanogaster group species, but it is perfectly conserved amongst these five genomes. (PDF) [file pgen.1002515.s008.pdf]
